# Transcriptome analysis identifies genes involved with the development of umbilical hernias in pigs

**DOI:** 10.1371/journal.pone.0232542

**Published:** 2020-05-07

**Authors:** Mayla Regina Souza, Adriana Mercia Guaratini Ibelli, Igor Ricardo Savoldi, Mauricio Egídio Cantão, Jane de Oliveira Peixoto, Marcos Antônio Zanella Mores, Jader Silva Lopes, Luiz Lehmann Coutinho, Mônica Corrêa Ledur

**Affiliations:** 1 Programa de Pós-graduação em Zootecnia, Centro de Educação Superior do Oeste, Universidade do Estado de Santa Catarina, UDESC, Chapecó, Santa Catarina, Brazil; 2 Embrapa Suínos e Aves, Concórdia, Santa Catarina, Brazil; 3 BRF S.A, Faxinal dos Guedes, Santa Catarina, Brazil; 4 Laboratório de Biotecnologia Animal, Escola Superior de Agricultura “Luiz de Queiroz”, Universidade de São Paulo, Piracicaba, São Paulo, Brazil; Panstwowy Instytut Weterynaryjny - Panstwowy Instytut Badawczy w Pulawach, POLAND

## Abstract

Umbilical hernia (UH) is one of the most frequent defects affecting pig production, however, it also affects humans and other mammals. UH is characterized as an abnormal protrusion of the abdominal contents to the umbilical region, causing pain, discomfort and reduced performance in pigs. Some genomic regions associated to UH have already been identified, however, no study involving RNA sequencing was performed when umbilical tissue is considered. Therefore, here, we have sequenced the umbilical ring transcriptome of five normal and five UH-affected pigs to uncover genes and pathways involved with UH development. A total of 13,216 transcripts were expressed in the umbilical ring tissue. From those, 230 genes were differentially expressed (DE) between normal and UH-affected pigs (FDR <0.05), being 145 downregulated and 85 upregulated in the affected compared to the normal pigs. A total of 68 significant biological processes were identified and the most relevant were extracellular matrix, immune system, anatomical development, cell adhesion, membrane components, receptor activation, calcium binding and immune synapse. The results pointed out *ACAN*, *MMPs*, *COLs*, *EPYC*, *VIT*, *CCBE1* and *LGALS3* as strong candidates to trigger umbilical hernias in pigs since they act in the extracellular matrix remodeling and in the production, integrity and resistance of the collagen. We have generated the first transcriptome of the pig umbilical ring tissue, which allowed the identification of genes that had not yet been related to umbilical hernias in pigs. Nevertheless, further studies are needed to identify the causal mutations, SNPs and CNVs in these genes to improve our understanding of the mechanisms of gene regulation.

## Introduction

Pig husbandry has become one of the most important activities in livestock production and increment in pig production in the last years has been observed [1,2]. However, at the same time that production has increased some physiological problems have emerged, causing economic losses and affecting animal welfare. The umbilical hernia (UH), an anatomic defect characterized by the protrusion of abdominal content through the umbilical ring, is one of the most frequent defects affecting pig production. UH prevalence in pigs ranges from 0.40 to 2.25%, varying according to the breed, farm and production system [3,4]. Animals affected with UH experience generally reduced performance, with low growth rates, poor feed conversion, low meat quality and pain and discomfort, which could also lead to death [5,6]. Although it is known that economic losses caused by umbilical hernia have a huge impact in the pig industry, it is really hard to find reports with their estimates. However, considering only the mortality rate caused by the four most prevalent defects (Splayleg, scrotal/inguinal hernia, umbilical hernia and atresia ani), economic losses over US$100 million annually dollar in the global pig industry have already been estimated [7].

The development of umbilical hernia can be caused by external factors, such as physical lesions, high pressure in the abdomen, inappropriate removal of the umbilical cord, infections and management [5]. Meanwhile, it has been observed that related animals with the same management practices could present different phenotypes, i.e., being normal or affected with hernias [5], indicating that a genetic factor is also present [8]. Moreover, the heritability estimate of 0.25 for UH in pigs [9] and 0.40 in cattle [10] reinforce the genetic component regulating this trait. However, the mode of inheritance and its etiology remain unclear.

Several quantitative trait loci (QTL) associated with UH have already been reported for pigs. In a search in the pig QTLdb (https://www.animalgenome.org/cgi-bin/QTLdb/SS/index - 11/02/2019), 54 QTLs for umbilical hernia have been found in several pig breeds, being located in chromosomes SSC1, SSC2, SSC3, SSC4, SSC6, SSC7, SSC8, SSC9, SSC10, SSC11, SSC13, SSC14, SSC15, SSC16 and SSC17 [11–14]. Ding et al. (2009) [11] performed the first genomic study with UH and found some chromosomic regions related to the appearance of this condition. More recently, a copy number variation (CNV) polymorphism on SSC14 was found to be related to UH [12] and a highly significant QTL for this trait was detected in Norwegian Landrace pigs also in SSC14 [13]. This QTL explained approximately 8.6% of the phenotypic variance for UH, and the LIF Interleukin 6 Family Cytokine (*LIF*) and Oncostatin M (*OSM*) genes were located within this QTL, being considered candidates for functional studies [13]. Recently, in a genome-wide association study (GWAS) with crossbred pigs, SNPs in the chromosomes SSC4, SSC6, SSC11 and SSC13 were associated with umbilical hernia. In these regions, novel genes: *TBX15* (T-box 15) and *WARS2* (tryptophanyl-tRNA synthetase 2) in SSC4, and *LIPI* (lipase I) and *RBM11* (RNA Binding Motif Protein 11) in SSC13, were identified as possible candidates to the UH development [15].

Although some genomic regions associated with UH have already been identified, no functional studies with the umbilical ring tissue were performed in pigs. Therefore, knowing the complexity of this disorder, in this study, we have sequenced the umbilical ring transcriptome of normal and affected pigs to discover genes and pathways involved with the development of umbilical hernia in a swine purebred line using RNA-Seq.

## Material and methods

### Animals and sampling

In this study, 10 unrelated Landrace purebred females from the same nucleus farm, with high sanitary status, located in Santa Catarina State, south of Brazil, with approximately 90 days of age were used in a case and control design. The UH occurrence in this line is around 1.7%. From those 10 animals, 5 were affected with umbilical hernia (with intestinal loops forming a herniary sac) and 5 were normal (with no malformations and coming from families with no history of hernias). For each affected animal, a contemporary normal pig was chosen. The animals were transported to the Embrapa Swine and Poultry National Research Center, located in Concórdia-SC, to be evaluated and necropsied. Euthanasia was performed by electrocution desensitization for 10 seconds, followed by bleeding, according to the practices recommended by the Embrapa Swine and Poultry National Research Center Ethics Committee on Animal Utilization, approved under protocol #011/2014. Necropsy was carried out to evaluate the existence of eventual problems that could compromise the accuracy of the data and for the correct characterization of the umbilical hernia phenotype. Tissue samples were collected from the umbilical ring of normal and umbilical hernia-affected animals. Samples were placed in 4% paraformaldehyde buffer for histopathological analysis and those for gene expression analysis were immediately frozen in liquid nitrogen and, subsequently, stored at -80 °C for further RNA extraction.

### Histological analyses of umbilical ring tissue

Tissues from the umbilical ring region of the herniated or non-herniated animals were dehydrated in a series of crescent ethanol concentration, diaphanized with xilol and embedded in paraffin. Tissue sections with 2 to 5 μm thickness were cut with an automatic microtome, stained by the hematoxylin & eosin method and analyzed with optical microscopy. The cell types were evaluated in a 10x eyepiece with 5x to 100x objectives, following a routine histopathological analysis.

### RNA extraction, library preparation and sequencing

For RNA extraction, about 100 mg of each sample were macerated in liquid nitrogen using 1 mL of Trizol (Invitrogen, USA), according to the manufacturer’s instructions. Next, 200 μL of chloroform was added, shaken vigorously for 15 seconds and incubated at room temperature for 5 minutes. Centrifugation at 11,000 xg at 4 °C for 15 minutes was performed and 600 μL of the aqueous phase were transferred to a new tube containing 600 μL of 70% ethanol. This volume was then added to an RNEasy mini silica column (Qiagen, Germany) following the manufacturer’s instructions. The quality and quantity of the total RNA were evaluated in Biodrop spectrophotometer (Biodrop, UK), 1% agarose gel electrophoresis and Agilent 2100 Bioanalyzer (Agilent Technologies; Santa Clara, CA, USA). Samples with 260/280 nm ratio above 1.9 on Biodrop and with RNA integrity number (RIN) greater than 8.0 were used for preparing the RNA-Seq libraries.

To prepare the libraries, 4μg of total RNA from each sample were used with the TruSeq Stranded mRNA Kit (Illumina, Inc.; San Diego CA, USA) following the manufacturer’s recommendation. The size of the libraries was evaluated in the Bioanalyzer obtaining an average size of 300 bp for each sample. After checking the concentration and size of the libraries, the paired-end (2x100 bp) RNA sequencing was performed in the Illumina HiSeq2500 equipment (Illumina, Inc.; San Diego CA, USA) at the Center for Functional Genomics of ESALQ/USP. All samples (normal and affected pigs) were placed and sequenced in the same lane.

### Quality control, assembly and differential expression analysis

For the quality control, the SeqyClean tool [16] was applied for the removal of short reads (<70bp), low quality reads (QPhred <24), adapter sequences, contaminants (phiX) and poly A/T tails. After, the sequences were mapped against the pig reference genome (Sus scrofa, assembly 11.1), available in the Ensembl database version 94 (www.ensembl.org), using the STAR program [17]. The reads counting was performed with the HTSeq-count program [18]. The EdgeR package [19] from R [20] was used for identifying differentially expressed (DE) genes between normal and UH-affected pigs. The significance threshold to declare genes as DE was set to a False Discovery Rate (FDR) ≤ 0.05 after multiple correction tests to reduce type I error, following the Benjamini and Hochberg (BH) method [21]. The Multi-Dimensional Scaling (MDS) plot was created with R using the LogFC values of each expressed gene in the umbilical ring tissue of the normal and UH-affected pigs. The heatmaps were generated with the plots package from R [20] using the expression data of each sample for each DE gene. The FASTQ files were deposited at the SRA database with BioProject number PRJNA445856 and biosample numbers: SAMN08801040, SAMN08801041, SAMN08801042, SAMN08801043, SAMN08801044, SAMN08801045, SAMN08801046, SAMN08801047, SAMN08801048 and SAMN08801049.

### Validation of DE genes using quantitative PCR (qPCR)

The qPCR analysis was used to confirm the results found in the RNA-Seq, using the same tissue samples from the normal and affected pigs. The RNA extraction was performed as previously mentioned and the cDNA synthesis was carried out using 3 μg of total RNA and the SuperScript III First-Strand Synthesis SuperMix kit (Invitrogen, USA) standard protocol. For validation, the following 12 DE genes were chosen according to their functions: Matrix metallopeptidase 13 (*MMP13*), Vitrin (*VIT*), Alkaline ceramidase 2 (*ACER2*), Molecule CD3D (*CD3D*), Galactin 3 (*LGALS3*), Fos proto-oncogene, AP-1 transcription factor subunit (*FOS*), collagen and calcium binding EGF domains 1 (*CCBE1*), Plakophilin 3 (*PKP3*), Epiphycan (*EPYC*), S100 calcium binding protein A2 (*S100A2*), Aggrecan (*ACAN*) and Microtubule associated protein 1 light chain 3 gamma (*MAP1LC3C*). In addition, ten candidate reference genes were tested to select the appropriate reference genes to be used in the qPCR analysis (Table 1), as described by [22]. The primers were designed in exon-exon regions using primer-blast tool [23] and their quality was evaluated in the NetPrimer program (http://www.premierbiosoft.com/NetPrimer) (Table 1). The qPCR reactions were performed in the Quantstudio 6 (Applied Biosystems, USA) with a final volume of 15 μL containing 1X GoTaq qPCR Master Mix (2x) (Promega), 0.13 μM of the forward and reverse primers and 2.0 μL of 1:10 diluted cDNA. Reactions were performed in duplicates, with cycling 95° for 2 minutes, 40 cycles for 15 seconds of 95°C and 60° for 30 seconds. Furthermore, negative control samples were included to detect contaminations.

**Table 1:** Primers for the 12 target genes and the 10 candidate reference genes used in qPCR analysis of the umbilical ring tissue in pigs.

Ensembl ID	Target genes	Chr.	Primer sequence (5’ to 3’)
**ENSSSCG00000001832**	*ACAN*	7	F:CAGGAGGGGTTGTGTTCCATTA
	Aggrecan		R:CCTCCTCGAAAGTCAGTGAGTAG
**ENSSSCG00000034213**	*ACER2*	1	F:AAGGAGGTGCGACAACGTG
	Alkaline Ceramidase 2		R:TAGGGGAAGTGGAAGGCAGAT
**ENSSSCG00000034214**	*CCBE1*	1	F:GGGGGACAAGTACCCCAATG
	Collagen and Calcium binding EGF domains 1		R:GGGAGCAGGGCAATCTTCTG
**ENSSSCG00000034215**	*CD3D*	9	F:CTCCCGAGTGAGCCCCTAT
	CD3d molecule		R:GATCCAGGATGCGTTTTCCCA
**ENSSSCG00000034216**	*EPYC*	5	F:CTGCTGTGACTGCCCCAA
	Epiphycan		R:TCGATCTCAGCTGGACCCAT
**ENSSSCG00000034217**	*FOS*	7	F:GTGAAGACCATGCCAGGAGG
	Fos proto-oncogene, AP-1 transcription factor subunit		R:TAGCTGGTCTGTCTCCGCTT
**ENSSSCG00000034218**	*LGALS3*	1	F:CCCCTTCTGGACCACTGAAT
	Galectin 3		R:TGTTGTCCTCGTTGAAGCGT
**ENSSSCG00000034219**	*MAP1LC3C*	10	F:TGGAAACAGCTGGAGGAATGAG
	Microtubule Associated Protein 1 Light Chain 3 gamma		R:CCTCTCTTCTGGTTGCTAAGCTC
**ENSSSCG00000034220**	*MMP13*	9	F:AAGAGCATGGAGACTTCTACCC
	Matrix Metallopeptidase 13		R:GGAGGAAAAGCATGAGCCAA
**ENSSSCG00000034221**	*PKP3*	2	F:GCAGACAATAAGCTGGCCCT
	Plakophilin 3		R:ATCCCTGTGACGTTCTTGCG
**ENSSSCG00000034222**	*S100A2*	4	F:ACAAGTACTCGGGCCAAGAAG
	S100 calcium binding protein A2		R:TTCTCCCCTACAAAGCTGGG
**ENSSSCG00000034223**	*VIT*	3	F:GTCGAAGCCACCCACACTG
	Vitrin		R:AAGTCAGGTTCCTCCCCCA
Ensembl ID	Candidate reference genes*	**Chr.**	**Primer sequence (5’ to 3’)**
**ENSSSCG00000023971**	*H3F3A*	10	F: CTTTGCAGGAGGCAAGTGAG
	H3 histone, family 3A		R: TGGCATGGATAGCACACAGG
**ENSSSCG00000027637**	RPL32	13	F: CAAAATTAAGCGGAACTGGCGG
	Ribosomal protein 32		R: GCACATTAGCAGCACTTCAAGC
**ENSSSCG00000015108**	*HMBS*	9	F: AGGATGGGCAACTCTACCTGA
	hydroxymethylbilane synthase		R: ATGGATGGTGGCCTGCATAG
**ENSSSCG00000017509**	*RPL19*	12	F: ACCGCCACATGTATCACAGTC
	ribosomal protein L19		R: TGTGCTCCATGAGAATCCGC
**ENSSSCG00000004489**	*EEF1A1*	1	F: CCGCCAGGACACAGGT
	eukaryotic translation elongation factor 1 alpha 1		R: TTCCCATCTCCGCAGCCT
**ENSSSCG00000003166**	*RPL13A*	3	F: CCAAGCAGGTACTTCTGGGC
	ribosomal protein 13A		R: GGCAGCATGCCTCGCA
**ENSSSCG00000011213**	*TOP2B*	13	F: AGAAGAGCTGCTGCTGAAAGG
	topoisomerase (DNA) II beta		R: TCCCCGTCATTTGTCACAGG
**ENSSSCG00000020686**	*SDHA*	16	F: TTGTACGGAAGGTCTCTGCG
	succinate dehydrogenase complex flavoprotein subunit A		R: GATGACTCCACGACACTCCC
**ENSSSCG00000006062**	*YWHAZ*	4	F: ATCAGATTGGGTCTGGCCCT
	tyrosine 3-monooxygenase/tryptophan 5-monooxygenase activation protein zeta		R: GGTATCCGATGTCCACAATGTC
**ENSSSCG00000016737**	*PPIA*	18	F: GCGTCTCCTTCGAGCTGTTT
	peptidyl-prolyl cis-trans isomerase A		R: ACTTGCCACCAGTGCCATTA

Chr: chromosome; F: forward; R: reverse;

*Lorenzetti et al. (2018).

To determine the stability of the ten candidate reference genes in the umbilical ring tissue for selecting the best gene(s) as normalizer(s) in the qPCR analyses, the endoGenes pipeline (https://github.com/hanielcedraz/endoGenes) was used. This pipeline performs an automated analysis of the BestKeeper [24], geNorm [25] and NormFinder [26] tools and ranks the most stable genes with the RankAggreg package from R [27].

The Ct means of the 12 evaluated target genes were obtained and normalized using the most stable reference genes selected based on the previous step. After data normalization, the log2FC (Log_2_ Fold Change) values obtained from both qPCR and RNA-Seq analyses were compared using the Pearson´s correlation analysis in the R program [20].

### *In silico* functional analysis

Functional annotation of DE was performed using DAVID 6.8 database (https://david.ncifcrf.gov/). The clustering of biological processes (BP) was performed in Revigo (http://revigo.irb.hr/). A gene network was constructed with the BP in Cytoscape v3.7.1 [28]. Furthermore, it was verified whether the DE genes were in QTL regions for umbilical hernia occurrence in pigs using the Pig QTLdb from the Animal Genome Database (http://www.animalgenome.org/QTLdb/app).

## Results

### Histological analysis

**Fig 1:**
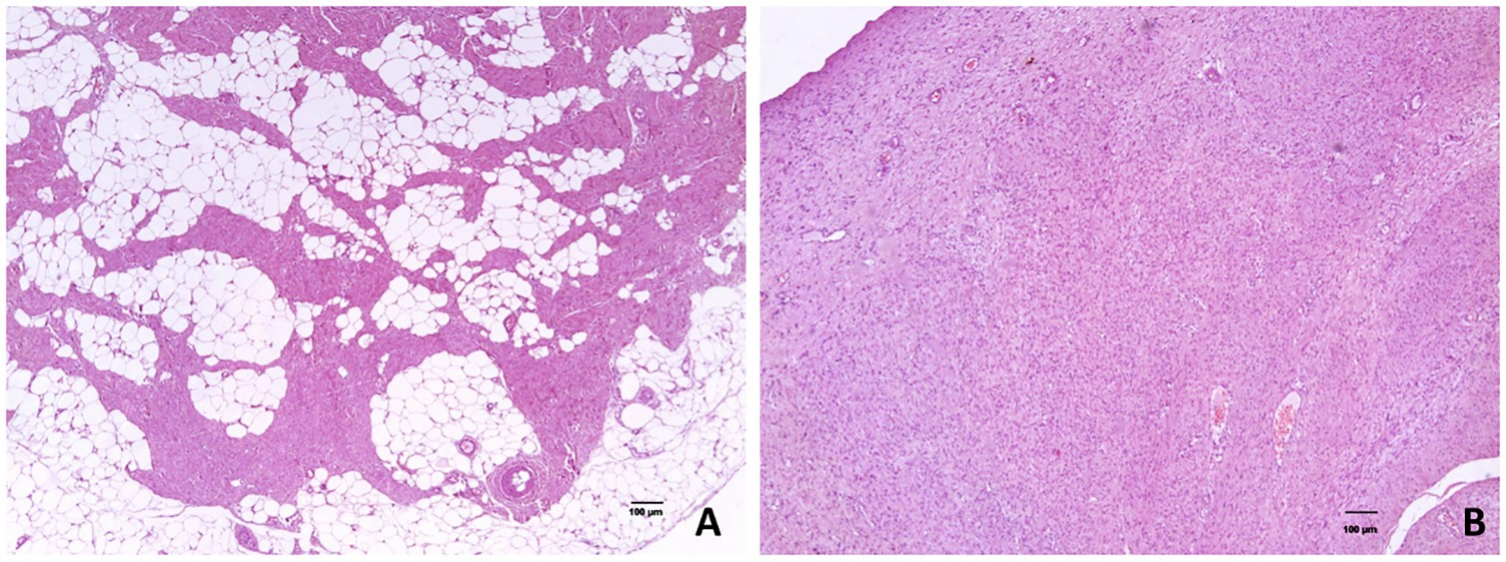
Hematoxylin & eosin stain histological section of the umbilical ring tissue sample from a normal (A) and an umbilical hernia-affected (B) piglet.

The histopathological evaluation has shown that, in general, the umbilical ring tissue of animals affected with umbilical hernia was thickened by an abundant proliferation of dense connective tissue. On the other hand, a normal amount of collagen fibers of connective tissue interspersed with adipose tissue was found in the umbilical ring tissue of the normal pigs (Fig 1).

### Sequencing and mapping

Sequencing of the umbilical ring tissue transcriptome produced about 24 million paired-end reads per sample (S1 Table). After the quality control, about 2.3 million reads were removed, remaining in average 21.7 million reads/sample (S1 Table). About 99.85% of the reads were mapped in the genome (Sus scrofa 11.1), with an average of 82% of the reads mapped in genes.

### Differentially expressed genes

**Fig 2:**
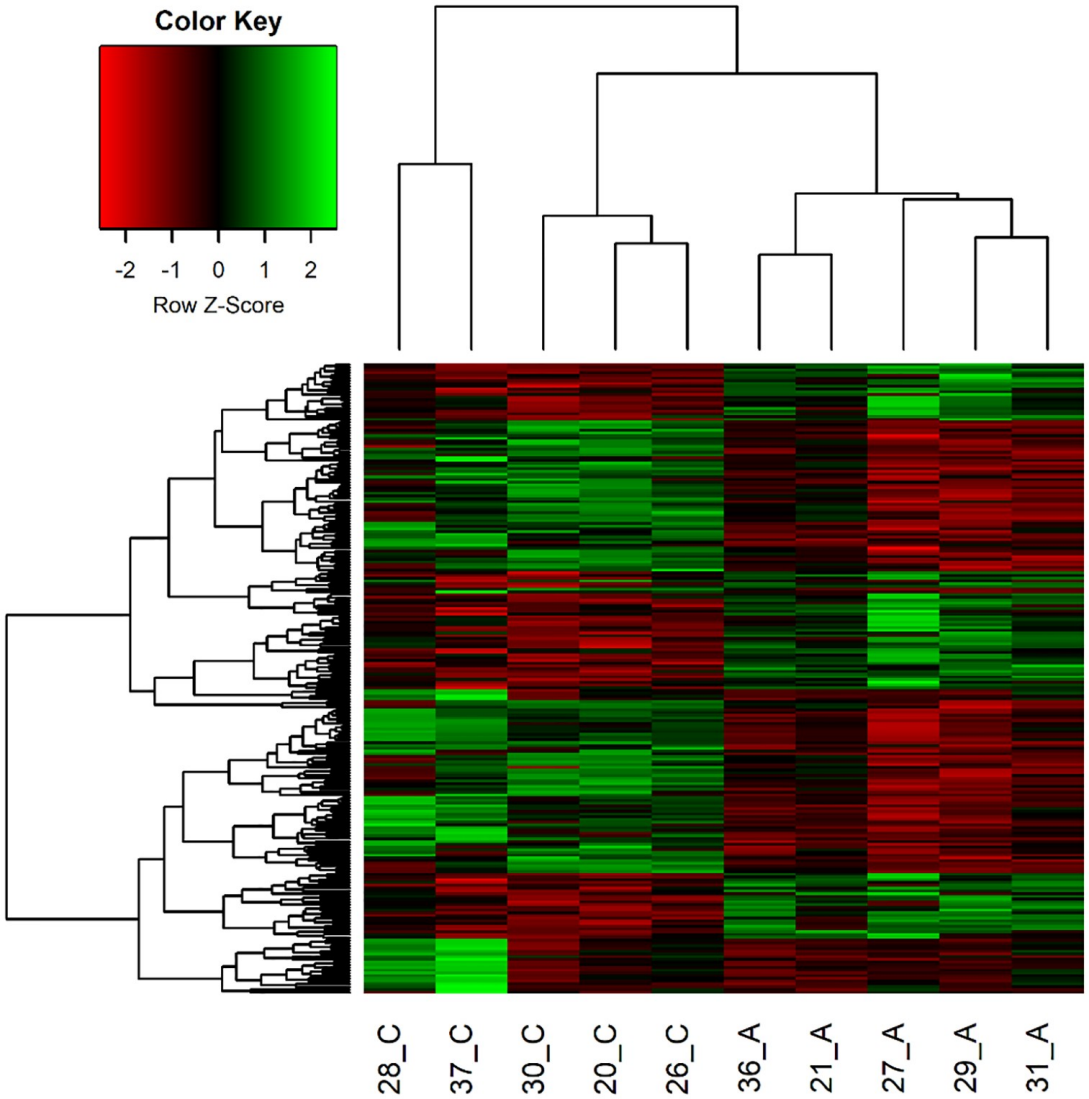
Heatmap with 230 differentially expressed genes between animals affected with umbilical hernia (21A, 27A, 27A, 29A, 31A and 36A) and normal piglets (20C, 26C, 28C, 30C and 37C). The expression for each gene is shown in the rows and samples are visualized in the columns, showing a hierarchical clustering of genes and samples. Genes are upregulated (in green) and downregulated (in red) in the affected samples.

From the 25,880 genes annotated in the swine genome (Ensembl 94), a total of 13,216 was expressed in the umbilical ring tissue. From those, 230 genes were DE, being 145 (63.04%) downregulated and 85 (36.96%) upregulated in animals affected with umbilical hernia compared to the normal pigs (S2 Table). A clear separation of samples from UH-affected and normal pigs was observed in both the heatmap (Fig 2) and the MDS plot (S1 Fig), comparing the groups of animals used in this study. Some variation between samples within group is expected (Fig 2) since the tissue evaluated is quite complex, especially in animals from the normal group, where the umbilical ring is not as apparent as it is in the affected pigs.

The 10 most down and upregulated genes based on the Log2FC of the UH-affected compared to normal pigs (Table 2) are mainly related to the organization of the extracellular matrix, morphogenesis, cartilage development and biosynthesis processes. Some of these transcripts identified in our study have not yet been characterized in Ensembl 94.

**Table 2:** Top 10 down and upregulated genes in the umbilical ring tissue of normal and umbilical hernia-affected piglets.

ENSEMBL ID	Gene symbol	Gene name	Log2FC
ENSSSCG00000037358	*SAA3*	Serum Amyloid A-3 Protein	-7,40
ENSSSCG00000014988	*MMP13*	Matrix Metallopeptidase 13	-7,31
ENSSSCG00000037009			-5,94
ENSSSCG00000036318			-5,75
ENSSSCG00000036203			-5,64
ENSSSCG00000004195	*ARG1*	Arginase 1	-5,53
ENSSSCG00000036127			-5,24
ENSSSCG00000040651			-5,21
ENSSSCG00000037141			-4,93
ENSSSCG00000036445	*CXCL13*	C-X-C Motif Chemokine Ligand 13	-4,85
ENSSSCG00000003509	*SH2D5*	SH2 Domain Containing 5	2,76
ENSSSCG00000016883	*ISL1*	ISL LIM Homeobox 1	2,78
ENSSSCG00000001832	*ACAN*	Aggrecan	2,81
ENSSSCG00000006021	*KCNV1*	Potassium Voltage-Gated Channel Modifier Subfamily V Member 1	2,99
ENSSSCG00000026780	*EDIL3*	EGF Like Repeats And Discoidin Domains 3	3,01
ENSSSCG00000003431	*NPPB*	Natriuretic Peptide B	3,05
ENSSSCG00000036566	*LY6G6C*	Lymphocyte Antigen 6 Family Member G6C	3,38
ENSSSCG00000038121	*TCHH*	Trichohyalin	3,76
ENSSSCG00000034838	*MAP1LC3C*	Microtubule Associated Protein 1 Light Chain 3 Gamma	3,95
ENSSSCG00000033927			4,26

### Selection of reference genes and confirmation of RNA-Seq results with qPCR

**Fig 3:**
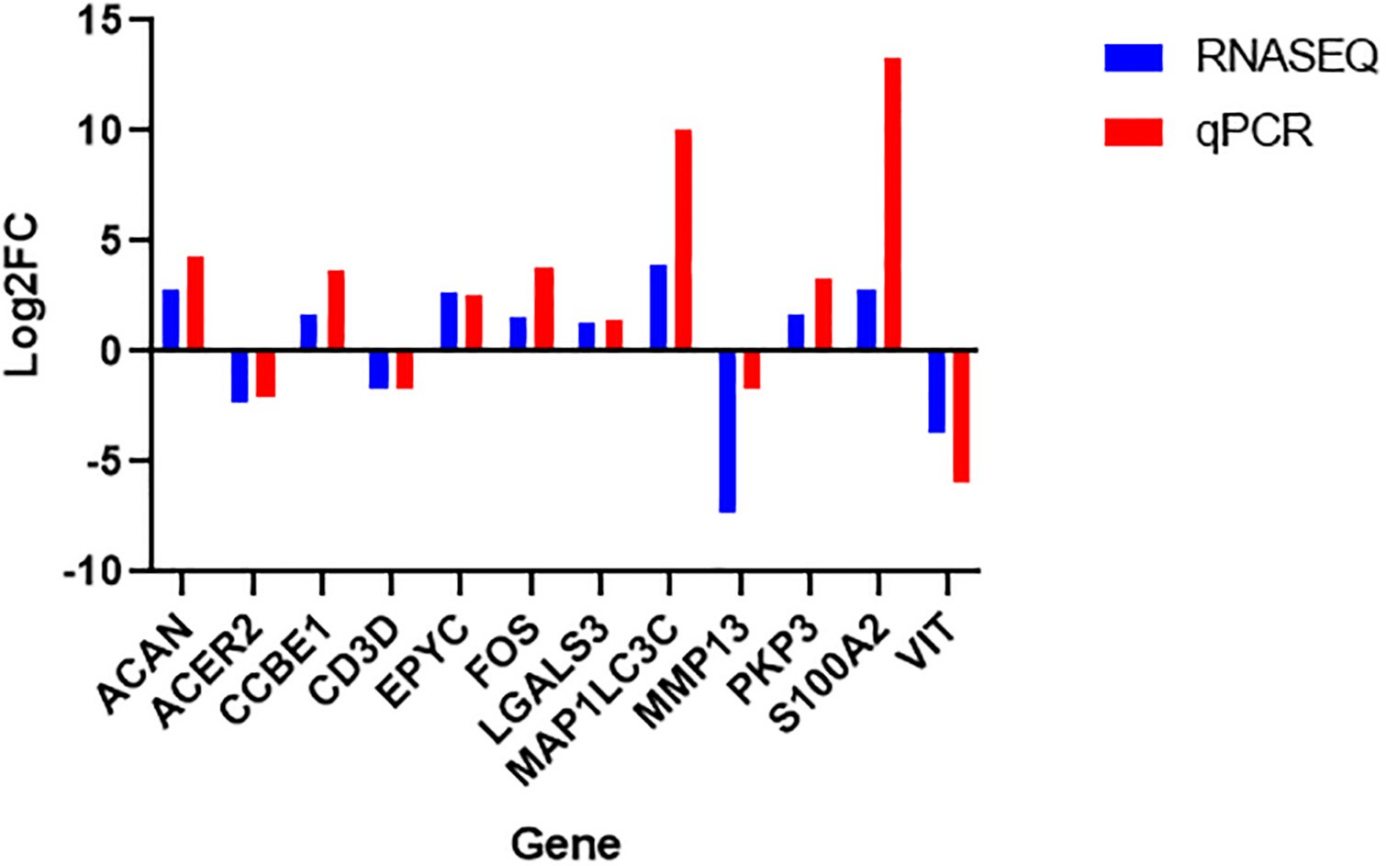
Comparison of Log2FC expressed values between the RNA-Seq and qPCR methodologies for the 12 target genes chosen for validation.

**Fig 4:**
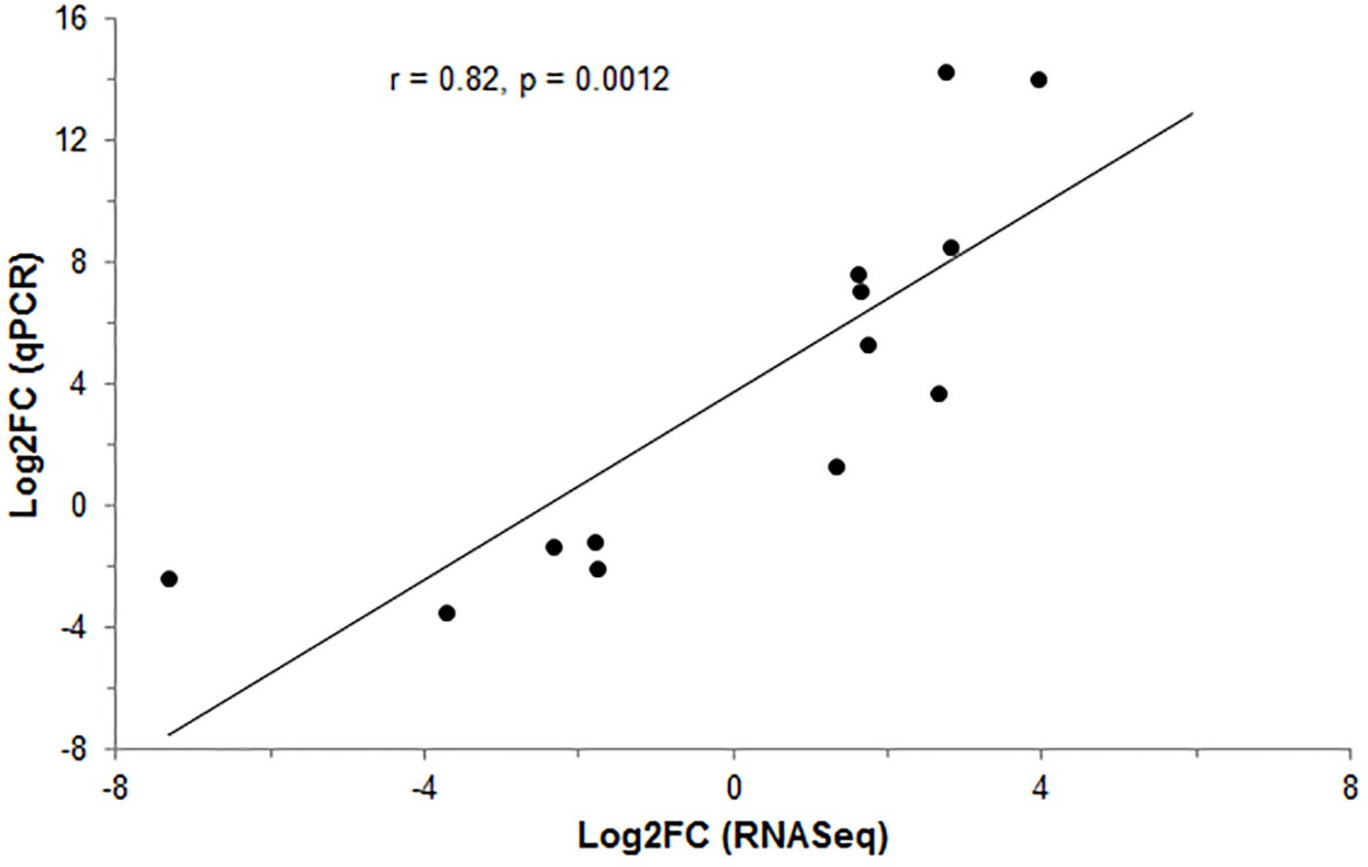
Pearson`s correlation (r) between Log2FC values of RNA-Seq and qPCR analyses for the 12 target genes selected for validation.

From the 10 candidate reference genes tested, H3 histone 172 family 3A (*H3F3A*) and Ribosomal protein 32 (*RPL32*) were considered the most stable and therefore used in qPCR normalization. The qPCR analysis confirmed the RNA-Seq results with a high concordance between the Log2FC of the RNA-Seq and the qPCR analysis (Fig 3). The pairwise correlation analyses between the Log2FC of the two methodologies showed that the results from RNA-Seq obtained in this study were consistent (r = 0.82, Fig 4).

### *In silico* analyses

**Fig 5:**
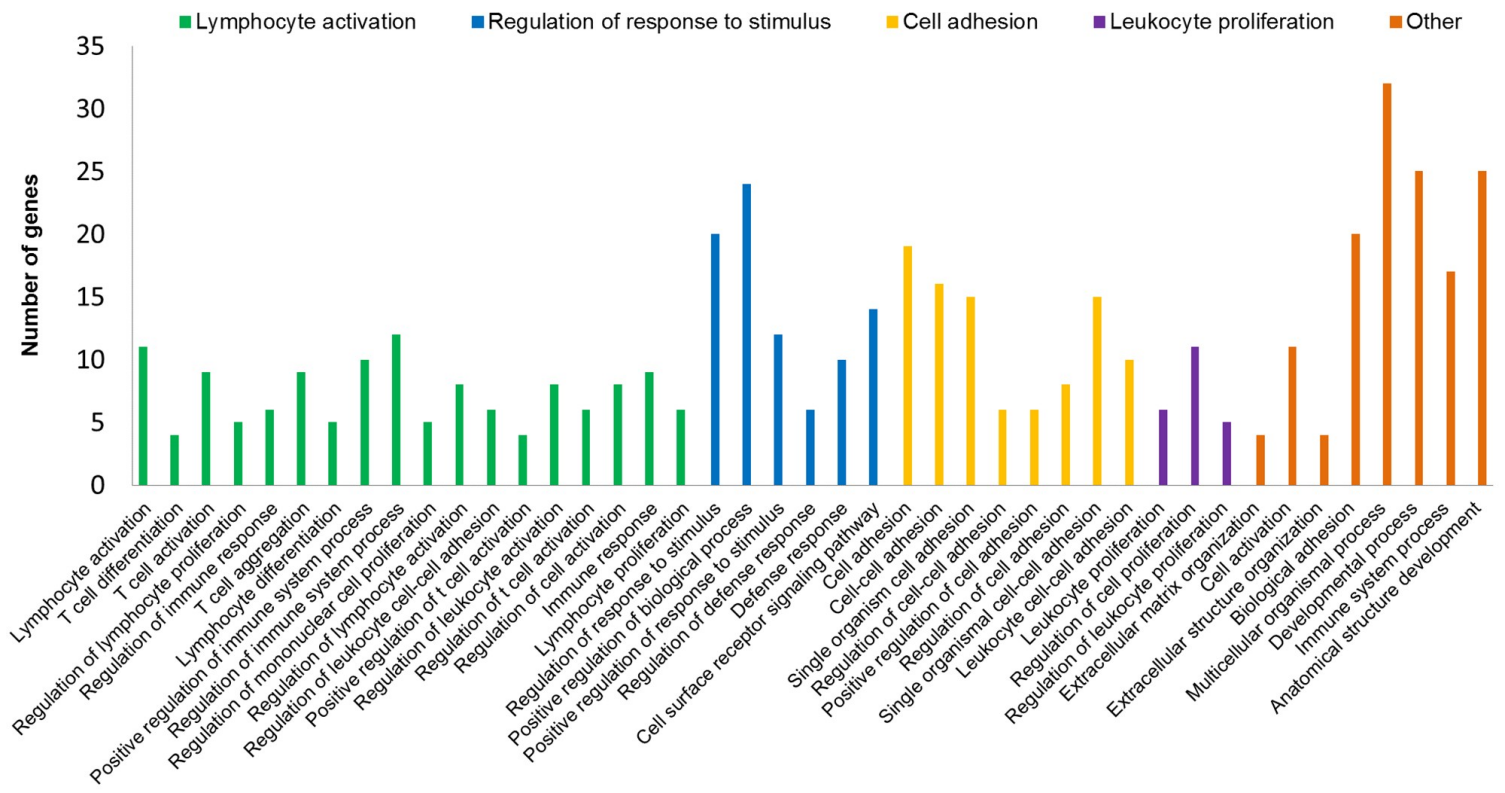
Significant biological processes of Differentially Expressed (DE) genes related to umbilical hernia in pigs. The X-axis shows the total number of genes that were DE in each biological process, based on the genetic ontology using the David 6.8.

**Fig 6:**
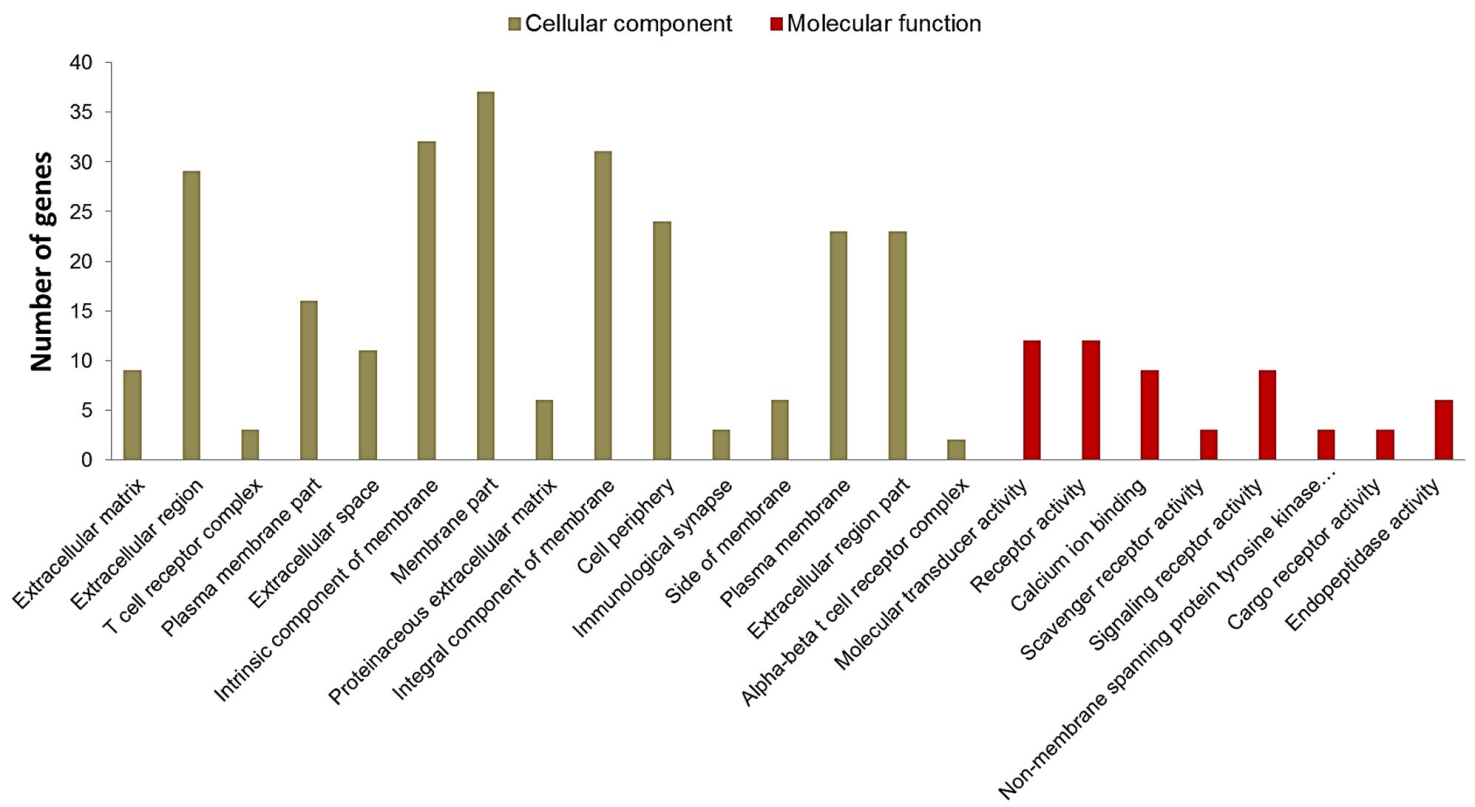
Significant cell components and molecular functions of Differentially Expressed (DE) genes related to umbilical hernia in pigs. The X-axis shows the total number of genes that were DE in each of them, based on the genetic ontology using the David 6.8 database.

From the 230 DE genes found in this study, 161 were selected for the *in silico* analyses considering the Log2FC interval from -1.5 to +1.5. From this set of genes, 91 were identified in David 6.8 database, comprising BP, molecular functions (MF) and cellular components (CC). A total of 68 significant BP (p <0.05) were identified, which were grouped using Revigo in 20 superclusters: cell adhesion, lymphocyte activation, extracellular matrix organization, cell activation, biological adhesion, regulation of cell proliferation, immune system, among others (Fig 5, S3 Table). The main cell components were involved in the cell matrix, T cells and cell membrane, comprising the molecular functions related to calcium activity, molecular transduction and receptor activity (Fig 6).

### Gene network

**Fig 7:**
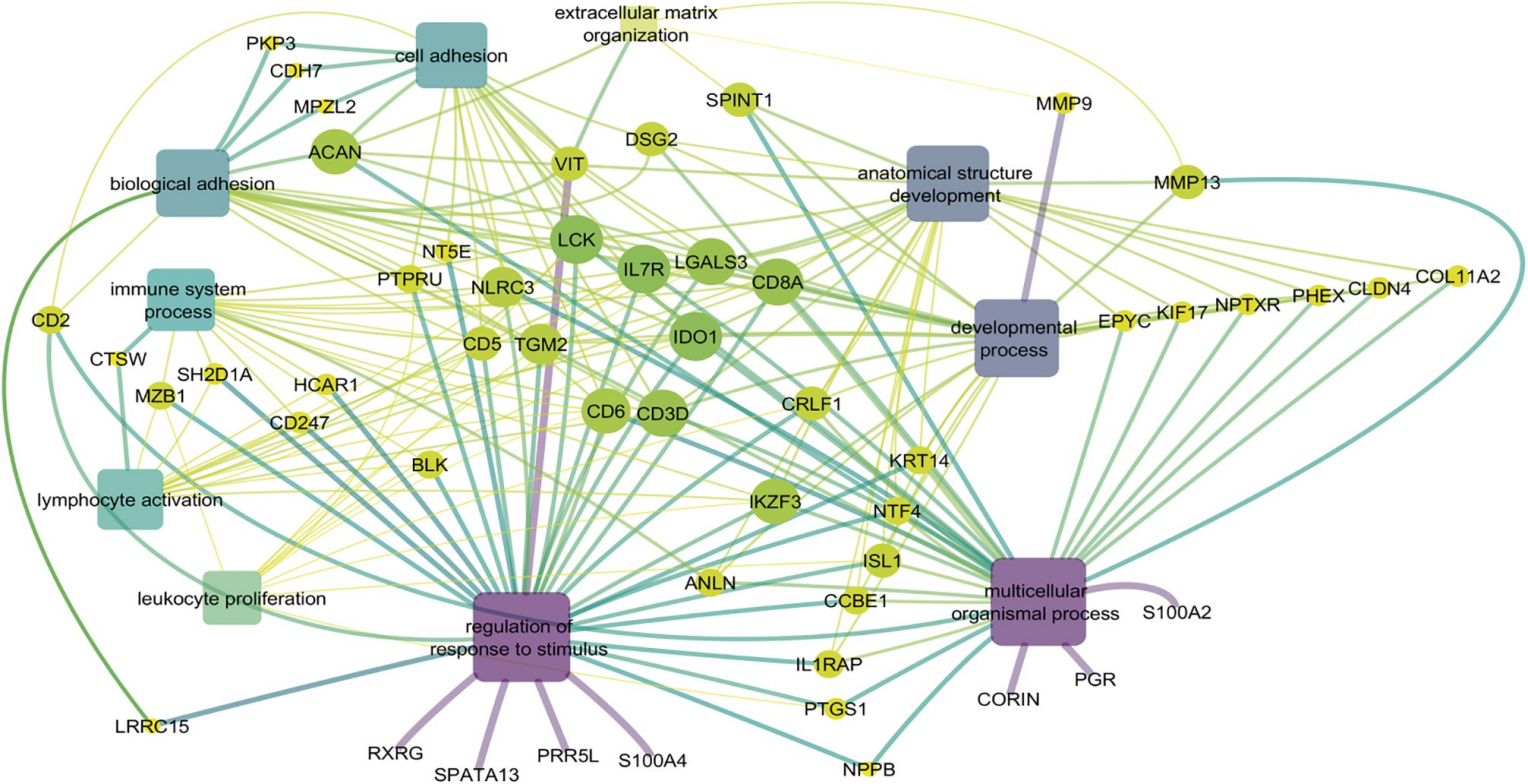
Gene network constructed with differentially expressed genes and main biological processes related to umbilical hernia using Cytoscape. Differentially expressed genes are visualized in the circles and biological processes in the rectangles. Node sizes indicate the number of predicted gene interactions. The edge colors indicate the betweenness of the edges (low values are in small size and in bright colors).

The constructed gene network (Fig 7) based on the DE genes, the BP and the cluster performed with Revigo showed the probable bioprocesses that would be the most involved with the umbilical hernia development. A set of 10 BP was selected for constructing the gene network: development of anatomical structure, biological and cellular adhesion, lymphocyte activation, leukocyte proliferation, extracellular matrix organization, development processes, and multicellular processes of the organism and regulation of stimulus responses. In this analysis, we were able to relate 53 genes distributed according to their interactions and importance in these processes. Furthermore, in Fig 7 it is possible to observe that several genes identified in the enrichment analysis are involved in various BP, since those related to development and cell adhesion until those related to immune processes, in which the later have not yet been associated with UH in pigs. This information can be seen, for example, for the genes ACAN, VIT and some MMPs that are connected and acting in several BP related to the DE genes.

### DE genes located in QTL regions for umbilical hernia

With the 230 DE genes, a query performed in the pig QTLdb [29] pointed out that only six of these genes were located in QTL regions previously associated to UH [29]. Four of them were mapped to SSC1: Alkaline ceramidase 2 (*ACER2*), Solute carrier family 2 member 6 (*SLC2A6*), Prostaglandin-endoperoxide synthase 1 (*PTGS1*) and *LGALS3*; one was located in SSC2: KN motif and ankyrin repeat domains 3 (*KANK3*) and the other in SSC7: FOS. Moreover, three other DE genes found in our study were located in QTL regions for scrotal/inguinal hernias: *ACAN* mapped in SSC7, Butyrylcholinesterase (*BCHE*) in SSC13 and *KANK3* in SSC2.

## Discussion

Some studies have been performed to identify the genetic factors involved in the development of umbilical hernias, and QTL regions, SNPs and candidate genes associated with the appearance of this defect have been detected [5,11–13]. In this study, using RNA-Seq, we sequenced the umbilical ring transcriptome to discover possible genes involved in the occurrence of umbilical hernias in pigs. Here, a total of 230 genes were DE between normal and UH-affected pigs (S2 Table), even considering the complexity of the tissue collection and the naturally individual response of the animals. Using these genes, it was possible to observe a similar expression profile within groups, in which the heatmap was able to identify the samples according to the previous group characterization, reinforcing the correct separation of the animals based on the phenotypic trait (Fig 2). Additionally, in the current study, the stability of 10 endogenous candidate genes in the inguinal ring tissue was evaluated since no specific reference genes were reported for this tissue in pigs to date. Therefore, the *H3F3A* and *RPL32* were the most reliable reference genes under this experimental condition to obtain accurate gene expression profiles in this complex tissue. Twelve of 230 DE genes in the RNA-Seq experiment were selected to be validated by qPCR, which has confirmed the consistence of our RNA-Seq findings (Figs 3 and 4). Since the etiology of umbilical hernias is not yet fully understood, identifying genes and biological processes involved in the development of this hernia is essential to find strategies to reduce this anomaly in pig production systems. From the 68 BPs found in our study, the following can be highlighted and will be further discussed: extracellular matrix, cell adhesion, development of the anatomical structure and immune system. This allowed a closer observation of the relationship of the DE genes with BPs in the organism.

### Extracellular matrix

The extracellular matrix (ECM) provides support and resistance to the tissues and organs of the whole body, acting in biochemical processes related to morphogenesis, differentiation and homeostasis of the tissues [30]. Also, in the ECM there are molecules responsible for adhesion, migration, proliferation, differentiation and cellular survival of the tissue [31]. This very organized structure is divided into molecules responsible for the formation of fibers (collagens, elastin and fibronectins) and the interfibrillar proteoglycans and glycoproteins bridges [32,33]. In the fiber-forming group, the collagens can be highlighted, since they are responsible for the development and tissue resistance, and regulation of cell adhesion [34]. In the interfibrillar group, we highlight the proteoglycans that act in several functions, mainly related to binding, hydration, transport and resistance to force [30].

Two BPs were associated with ECM and both had the five clustered genes: *ACAN*, *MMP13*, Matrix metallopeptidase 9 (*MMP9*), Serine peptidase inhibitor, kunitz type 1 (*SPINT1*) and *VIT*. In addition, five cellular components related to matrix were found (Fig 6) and the most enriched genes were *ACAN*, *SPINT1*, *VIT*, *MMP13*, *MMP9*, Collagen type XI alpha 2 chain (*COL11A2*), *CCBE1*, *LGALS3*, Collagen type VI alpha 5 chain (*COL6A5*), cathepsin W (*CTSW*), S100 Calcium binding protein A4 (*S100A4*) and *EPYC*. The association of these genes in ECM bioprocesses and their location in extracellular matrix-related cell component (CC) reinforce their relationship with structural support and important biochemical signals in the cells and tissues of animals [35]. The identified genes involved in the maintenance of ECM clarify the relationship between ECM and umbilical hernias. It is known that problems in the connective tissue, such as disturb in collagen production, have already been associated with the appearance of hernias [36]. Furthermore, in the gene network it was possible to group genes that were enriched in ECM bioprocesses (Fig 7), such as *ACAN*, *MMP13*, *MMP9*, *SPINT1* and *VIT*, which were DE in our work.

Matrix metallopeptidase (*MMP*) family members are directly bound to collagen degradation and regulation. The *MMP13* gene is responsible for the degradation of type II collagen in cartilage and *MMP9* degrades type VI collagen [37,38]. In the present study, the *MMP13* and *MMP9* genes were 7.3 and 3.9 times less expressed in animals affected by umbilical hernia than in normal animals. The downregulation of these genes may be related to tissue disorders due to their role in collagen production. Also, three other genes of the collagen family were downregulated in the affected pigs: *COL6A5* (-4,3); *COL11A2* (-3.7) and Collagen type II Alpha 1 chain (*COL2A1;* -3.4). The collagen family genes are involved with the production of fibers, structural organization and strength to the connective tissues in the animal organism [39,40]. The relationship of metallopeptidase genes, such as *MMP1* and *MMP13*, with inguinal hernias in humans has already been verified [41]. Moreover, *MMP2*, *COL2A1*, *COL1A2* and *COL1A1* genes were associated to scrotal herniation in pigs [42]. In addition, mutations in *COL6A1*, *COL6A2* and *COL6A3* genes were considered causal mutations for congenital muscular dystrophy, a disease that affects connective and muscular tissue in humans [43]. Zhang et al. (2002) [44] reported that mutations in collagen genes *COL6A1*, *COL6A2* and *COL6A3* led to their mRNA decay. Moreover, Sabatelli et al. (2012) [45], demonstrated that *COL6A5* plays a key role in the tensile stress of connective tissue. Tagliavini et al. (2014) [46], verified that a defect in the *COL6A6* gene might contribute to collagen-related disorders.

There are several studies addressing MMP and collagen families as responsible for problems in the tissue and also for the manifestation of several types of hernias in different species [40,42–44,46]. Our findings are similar to those already reported, indicating that those genes related to ECM might be possibly triggering umbilical hernia in pigs. Thus, the downregulation of this set of genes could lead to problems related to the production of collagen, consequently causing tissue weakness and injury and, eventually, the formation of umbilical hernia.

### Cell adhesion

The cell adhesion BP is highly related to biological adhesion (Fig 7), both allowing cellular connections and binding to organisms or substrate. In particular, cell adhesion is directly linked to adhesion to the extracellular matrix [33], tissue development and maintenance, cell differentiation, migration, communication, regulation and survival [47,48]. In our study, 19 DE genes were grouped in this BP: *ACAN*, molecule CD2 (*CD2*), *CD3D*, molecule CD5 (*CD5*), molecule CD6 (*CD6*), molecule CD8A (*CD8A*), cadherin 7 (*CDH7*), desmoglein 2 (*DSG2*), indoleamine 2, 3-dioxygenase 1 (*IDO1*), interleukin 7 receptor (*IL7R*), Lymphocyte Cell-Specific Protein-Tyrosine Kinase (*LCK*), *LGALS3*, myelin protein zero like 2 (*MPZL2*), NLR Family Contingency Domain 3 (*NLRC3*), 5’-nucleotidase ecto (*NT5E*), *PKP3*, protein tyrosine phosphatase, type U receptor (*PTPRU*), transglutaminase 2 (*TGM2*) and *VIT* (Fig 7, S3 Table).

The *ACAN* gene belongs to the aggrecan proteoglycan family and was 2.8 times more expressed in the hernia-affected animals than in the normal pigs (Table 1). This gene has an important role in the cell adhesion process of the matrix, providing integrity, binding and resistance to cartilaginous tissue [49]. Polymorphisms in the *ACAN* have already been associated with hernias and cartilage degeneration [50,51]. However, there are no studies on the levels of *ACAN* gene expression for comparison. The observed *ACAN* upregulation in UH-affected pigs can lead to an exacerbated production of collagen. Association between abnormal amount of collagen and herniation was already reported [52]. Moreover, the *ACAN* has also been associated with other types of hernia, such as disc hernia [53].

The *VIT* gene was 3.7 times downregulated in the affected animals (S2 Table). *VIT* is responsible for encoding a protein related to the ECM and also participates in cell adhesion and cell migration [54,55]. The vitrin protein is similar to proteins that participate in the neural development and in the integrity of the extracellular matrix [56–58]. In the intact human cartilaginous tissues, higher levels of *VIT* were observed, whereas in tissues with cartilage problems the expression of this gene was reduced [59]. The *VIT* downregulation detected in the present study may disturb the production of cell adhesion proteins, reducing the integrity of the umbilical ring making this tissue more susceptible to hernia occurrence.

Two other genes were clustered only in the cellular and biological adhesion processes: Cadherin 7 (*CDH7*) and Leucine Rich Repeat Containing 15 (*LRRC15*), being, respectively, 2.5 and 1.8 times upregulated in the affected group compared to the normal pigs (S2 Table). The *CDH7* gene is involved in the cell structural and functional organization in various tissues [60]. Cadherins have a very important role in the cell adhesion process promoting cell binding, and mutations in these genes were associated with delayed growth and development [61,62]. The *LRRC15* gene is involved with cellular interactions, acting mainly on cell adhesion and on cell-cell and extracellular matrix interactions [63]. In animals, the actions of *LRRC15* are still poorly understood, however, in humans, cancer-damaged tissues had higher expression of this gene than normal tissues [64]. Possibly, the imbalance in the expression of the genes grouped in those BPs can interfere in tissue remodeling, causing disorders related to muscular fibers, weakening the umbilical ring tissue and favoring the occurrence of umbilical hernias in pigs.

Furthermore, another process that can affect cell adhesion, cell death and ECM BP is the autophagy, which is involved in cellular degradation to promote cell homeostasis [65]. One of the genes involved with autophagy is the *MAP1LC3C*, which was highly expressed in the affected when compared to the normal pigs in both RNA-Seq and qPCR experiments (Fig 3). This gene acts preventing the apoptosis, being regulated by different signaling pathways, including those calcium dependent, helping to maintain the cell fate and, consequently, the normal physiology [66]. The upregulation of *MAP1LC3C* has already been observed in pigs with scrotal hernia [67]. Also, the expression of *MAP1LC3B*, another gene from the *MAP1LC3* family, was increased in humans with incisional hernia (IH), concomitantly with the enhancing of apoptosis signaling, affecting the cell death and ECM detachment process, which contributed to the appearance of hernias in the fascia of patients with IH [68].

### Development of anatomical structure

Twenty-five DE genes were grouped in the development of anatomical structure BP (Fig 5, S3 Table). Furthermore, 11 of them participate in extracellular matrix CC and four enriched molecular functions related to calcium ion bonds (Fig 6). From this set of genes, we highlight *KRT14* (Keratin 14), *CCBE1*, *ACAN*, Desmoglein 2 (*DSG2*) and *EPYC*, which were more enriched in the anatomic development process in the gene network (Fig 7) and were upregulated in animals affected by umbilical hernia. The *KRT14* gene encodes proteins from the keratin family, which are structural proteins that provide skin resistance and elasticity [69,70]. Studies in humans have identified mutations in this gene being responsible for different skin diseases [69–72]. Other genes involved with the cell development are calcium binding proteins, *S100A2* and *S100A4*, and *PKP3* genes, which were also upregulated in the UH affected group. These genes are involved in cell differentiation and cell cycle, acting in epithelial tissues and skin [73,74] and, although there is no information regarding these gene functions in the UH development, they could maintain the balance between cell growth and differentiation with apoptosis.

The *EPYC* gene, previously known as Dermatan Sulfate Proteoglycan 3 (*DSPG3*), is predominantly expressed in cartilaginous tissues [75,76]. *EPYC* has main functions in the fibrilogenesis, which is characterized by the development and regulation of collagen fibrils in the embryonic period [77]. Tajima et al. (1999) [78], demonstrated that deficiency in the *EPYC* gene could cause Ehlers-Danlos syndrome in Dutch breed calves. This hereditary syndrome causes a defect in the connective tissue, due to changes in collagen synthesis and/or assembly of the collagen structure [79–81]. Due to the problems in the fibrillar collagen, the skin becomes fragile and with high risk of rupture [81]. Several studies have associated this syndrome with defects in collagen production [82], however, the actual function of the *EPYC* is not yet well known in pigs.

Two other gene, *DSG2* and *CCBE1*, were enriched in the anatomical structure BP and grouped in the calcium ion binding MF. The *DSG2* gene belongs to the desmoglein family, which are important components of the cadherins that integrate the desmosomes [83]. Desmossomes are characterized as structures of cell-to-cell linkages, which provide mechanical stability [83,84] and are crucial for embryonic development and tissue integrity [85]. Moreover, *DSG2* is expressed in many tissues and participates in calcium binding and cell adhesion [84]. The *CCBE1* gene encodes proteins responsible for the remodeling and migration of the extracellular matrix and is directly related to the calcium and collagen [86,87]. In humans, studies have shown that mutations in the *CCBE1* gene are associated with genetic problems, including the occurrence of umbilical and inguinal hernias [88–90]. Thus, the differential expression of these genes related to the development of the anatomical structure is a strong indication that alterations in this BP can cause deregulations or modifications in the structure of the tissue. These findings are reinforced by the histological changes observed in the umbilical ring tissue between the normal and UH-affected pigs. The umbilical ring of animals affected with umbilical hernia was thickened by an abundant proliferation of dense connective tissue, while a normal amount of collagen fibers of connective tissue interspersed with adipose tissue was found in the umbilical ring region of the normal pigs (Fig 1).

### Immune system

In this study, several DE genes enriched BP related to the immune system, where most of them were downregulated in the affected animals (S3 Table). Also, the gene network indicated three significant BP: lymphocyte activation, leukocyte proliferation and immune system process (Fig 7). These three similar processes grouped 21 genes, of which 11 were grouped in membrane CC and six of them in molecular function of transduction activity (Figs 5 and 6). Here, the cluster of differentiation (CD) gene family were highly represented by the *CD2*, *CD3D*, *CD5*, *CD6*, *CD247* and *CD8A* genes, that were downregulated in UH-affected animals compared to the normal pigs. These CD genes are directly linked to other biological processes, such as regulation of response to stimuli, development of anatomical structure, cell adhesion, and developmental processes (Fig 7). For instance, the *CD3D* gene is responsible for encoding a T cell receptor protein and performing signal transduction [91]. Another gene of this family, *CD34*, was downregulated in humans affected by inguinal hernias [92]. However, information about these genes are scarce in the literature and here is the first time that these genes are being associated with umbilical hernia in pigs.

The *EDIL3* (EGF as repeats and discoid domains 3) is another immune-related gene and was 3 times upregulated in affected animals compared to normal pigs (S1 Table). Studies have indicated that when there is injury, epithelial cells, macrophages and fibroblasts produce growth factors such as epidermal growth factor (EGF) and transforming growth factor (TGF) to prevent the problem [93]. When this occurs, there is an increase in the epithelial-mesenchymal transition, which is responsible for the healing, regeneration and fibrogenesis of the tissue [94–96]. However, when the animal has a disease that cannot be controlled, it becomes chronic and may result in an increase in the expression of EGF, fibronectin and proteoglycans [97]. In our study, the upregulation of genes responsible for the production of EGF (*EDIL3*) and proteoglycans (*ACAN* and *EPYC*) indicates that this expression profile is due to the advanced process of irreversible tissue degradation, where tissue repair genes are no longer active [98,99], and the *EDIL3*, *ACAN* and *EPYC* genes are over expressed producing high levels of proteoglycans and fibronectin. This may account for the accumulation and increased proliferation of dense connective tissue in pig tissue samples affected with umbilical hernia as observed in the histopathology (Fig 1). Thus, the downregulated genes clustered in the immune system BP are probably a consequence of the umbilical hernia, while the upregulated ones are possibly involved in the cause of the histological changes of the UH-affected tissue.

### Genes located in QTL regions

Ding et al. (2009) [11] were the first to identify regions related to the occurrence of umbilical hernias in pigs. Afterwards, other studies also identified regions related to umbilical hernias in pigs [5,12,13]. Six of the genes DE in our study were mapped to QTL regions already described in the literature for umbilical hernias in pigs: *ACER2*, *SLC2A6*, *PTGS1*, *LGALS3*, *KANK3* and *FOS*. These genes have very distinct functions, such as cell proliferation and survival (*ACER2*) [100], regulation of prostaglandin (*PTGS1*) [101] and glucose transport (*SLC2A6*) [102].

The *LGALS3* gene is part of membrane CC and was enriched in the gene network due to its involvement in several BP (Fig 7). Besides harboring a QTL region for umbilical hernia, *LGALS3* differential expression profile was confirmed by the qPCR methodology. This gene encodes a protein located in the extracellular matrix that acts in cell growth, survival, migration and adhesion [103]. *LGALS3* is also involved in the cellular apoptosis and innate immunity [104,105]. Another gene found in a QTL region for umbilical hernia is the *FOS*, which was upregulated in affected pigs and validated by qPCR. The *FOS* gene has a role in survival, proliferation, differentiation and cell death, organogenesis and stress response [106]. Studies with cancer patients have shown that *FOS* upregulation was correlated with the increase in cell death [107,108].

Furthermore, three other genes were located in QTL regions already identified for scrotal/inguinal hernias [11,109]: *ACAN*, *BCHE* and *KANK3*. The *ACAN* gene, which has been previously mentioned, related to cell adhesion and extracellular matrix [49], has been pointed out as a potential gene involved in the occurrence of hernias. The localization of *ACAN* in the QTL region for scrotal/inguinal hernias suggests a pleiotropic effect of this gene, being also involved in the manifestation of umbilical hernia.

The *KANK3* gene was downregulated in the affected animals and has been mapped to a QTL region for umbilical hernia and for scrotal/inguinal hernia [11]. This indicates a possible pleiotropic effect of *KANK3* in the manifestation of various types of hernias in pigs. Genes from *KANK* family (*KANK1* and *KANK2*) have already been related to the polymerization of actin filaments, fiber formation and cell migration [110]. These genes drive many cellular processes [111], especially those of transport and muscle contraction. This action may be related to the problem of umbilical hernia, since actin filaments are an important part of the body and especially of the muscle [112]. Furthermore, actin polymerization together with that of calcium is a key part of the adhesion process of epithelial cells [113]. Therefore, *KANK3* become a strong functional candidate to the development of umbilical hernia in pigs.

The umbilical hernia is considered a body wall defect, characterized by a body wall dysplasia, midgut protrusion in the umbilical ring, intact skin, normal umbilical cord and thinner wall possibly caused by the increased cell death [114,115]. This condition is very complex since it could be congenital or acquired, and in humans, they are characterized in infantile, when there is no obliteration of umbilical cord structures, or in adult, usually considered acquired [116]. Through the characterization of the umbilical ring transcriptome of normal and UH-affected pigs, a set of DE genes was prospected, where several BP and molecular functions possibly related to the herniation process were identified. The main biological processes involved with umbilical hernia were related to extracellular matrix, immune system, anatomical development, cell adhesion, membrane components, receptor activation, calcium binding and immune responses. Although there are few studies addressing the etiology of umbilical hernia in pigs and even in humans, it was possible to find several studies evaluating the development of human incisional hernia (IH) in fascia [68,117,118]. This is an acquired condition, which occurs after some types of surgeries. However, similar mechanisms have been described between IH and those found in our study with UH, such as unbalance of apoptosis, cell proliferation and migration, with similar MMPs and collagen genes altered [68,117,118]. Furthermore, disruptions in ECM functions and triggering of inflammation mechanisms were also described as contributing to hernia formation [68,117,118], corroborating with the BP enriched in this study. Therefore, knowing that samples from divergent phenotypes were collected, considering the family history, it is possible to highlight that the genes, such as *ACAN*, *MMPs*, *COLs*, *EPYC*, *VIT*, *LRRC15*, *CCBE1* and *LGALS3* can be considered strong candidates for the development of umbilical hernia in pigs and in other mammals”.

## Conclusions

We have generated the first transcriptome of the pig umbilical ring tissue, which allowed the identification of several genes that had not yet been related to umbilical hernias in pigs. The results pointed out *ACAN*, *MMPs*, *COLs*, *EPYC*, *VIT*, *CCBE1* and *LGALS3* genes as strong candidates to trigger umbilical hernias in pigs, because they are involved in hernia related biological processes since the embryogenesis. Nevertheless, further studies are needed to identify the causal mutations, improving our understanding of gene regulation and identifying alleles related to this defect to be used in animal selection to reduce the occurrence of umbilical hernia in pig production systems.

## Supporting information

S1 Table Reads number per samples and reads kept after quality control. (DOCX)

S2 Table List of 230 differentially expressed genes between normal and umbilical hernia- affected piglets. (DOCX)

S3 Table Main biological processes of genes differentially expressed between normal and umbilical hernia-affected piglets. Genes in bold are upregulated in the affected pigs.
(DOCX)

S1 Fig Multi-Dimensional Scaling (MDS) plot to visualize the separation between the five normal and the five umbilical hernia-affected piglets. (TIF)
