# Supplementary material for: Transcriptome analysis identifies genes involved with the development of umbilical hernias in pigs
Source: PLoS One. 2020 May 7;15(5):e0232542. doi: 10.1371/journal.pone.0232542 (PMC7205231; doi:10.1371/journal.pone.0232542)
Supplement: S1 Table — (DOCX) [file pone.0232542.s001.docx]

**Table S1. Reads number per samples and Reads kept after quality control**

| **Samples** | **Reads** | **Reads Kept after QC** |
| --- | --- | --- |
| **HE30** | 25,961,986 | 23,654,175 |
| **HE28** | 23,343,016 | 21,288,093 |
| **HE26** | 24,533,491 | 22,141,755 |
| **HE20** | 25,366,332 | 22,976,671 |
| **HE37** | 22,575,346 | 18,964,907 |
| **HE31** | 26,038,094 | 23,694,045 |
| **HE29** | 23,058,784 | 21,029,810 |
| **HE27** | 23,136,681 | 21,176,732 |
| **HE21** | 23,233,135 | 21,110,837 |
| **HE36** | 23,225,142 | 21,226,045 |
