# Supplementary material for: Transcriptome analysis identifies genes involved with the development of umbilical hernias in pigs
Source: PLoS One. 2020 May 7;15(5):e0232542. doi: 10.1371/journal.pone.0232542 (PMC7205231; doi:10.1371/journal.pone.0232542)
Supplement: S2 Table — (DOCX) [file pone.0232542.s002.docx]

**S2 Table:** List of 230 differentially expressed genes between normal and umbilical hernia- affected piglets.

| **Gene_stable_ID** | **logFC** | **FDR** | **Name** | **Gene_Description** |
| --- | --- | --- | --- | --- |
| ENSSSCG00000037358 | -7,40 | 0,011 | *SAA3* | Serum Amyloid A-3 Protein |
| ENSSSCG00000014988 | -7,31 | 0,012 | *MMP13* | Matrix Metallopeptidase 13 |
| ENSSSCG00000037009 | -5,95 | 0,001 |  |  |
| ENSSSCG00000036318 | -5,76 | 0,003 |  |  |
| ENSSSCG00000036203 | -5,64 | 0,003 |  |  |
| ENSSSCG00000004195 | -5,53 | 0,014 | *ARG1* | Arginase 1 |
| ENSSSCG00000036127 | -5,24 | 0,002 |  |  |
| ENSSSCG00000040651 | -5,21 | 0,010 |  |  |
| ENSSSCG00000037141 | -4,94 | 0,001 |  |  |
| ENSSSCG00000036445 | -4,85 | 0,002 | *CXCL13* | C-X-C Motif Chemokine Ligand 13 |
| ENSSSCG00000034766 | -4,83 | 0,002 |  |  |
| ENSSSCG00000037981 | -4,51 | 0,002 | *CNTNAP2* | Contactin Associated Protein Like 2 |
| ENSSSCG00000037214 | -4,49 | 0,037 |  |  |
| ENSSSCG00000021006 | -4,39 | 0,002 |  |  |
| ENSSSCG00000011186 | -4,38 | 0,002 | *COL6A5* | Collagen Type VI Alpha 5 Chain |
| ENSSSCG00000036224 | -4,33 | 0,013 |  |  |
| ENSSSCG00000039102 | -4,22 | 0,036 |  |  |
| ENSSSCG00000031292 | -4,19 | 0,002 |  |  |
| ENSSSCG00000039146 | -4,15 | 0,007 |  |  |
| ENSSSCG00000031037 | -4,00 | 0,010 |  |  |
| ENSSSCG00000006719 | -4,00 | 0,002 | *HSD3B1* | Hydroxy-Delta-5-Steroid Dehydrogenase, 3 Beta- And Steroid Delta-Isomerase 1 |
| ENSSSCG00000007436 | -3,95 | 0,003 | *MMP9* | Matrix Metallopeptidase 9 |
| ENSSSCG00000036990 | -3,89 | 0,000 | *DIO3* | Iodothyronine Deiodinase 3 |
| ENSSSCG00000032582 | -3,86 | 0,027 |  |  |
| ENSSSCG00000039111 | -3,77 | 0,042 |  |  |
| ENSSSCG00000008501 | -3,73 | 0,043 | *VIT* | Vitrin |
| ENSSSCG00000001473 | -3,72 | 0,002 | *COL11A2* | Collagen Type XI Alpha 2 Chain |
| ENSSSCG00000033417 | -3,56 | 0,042 |  |  |
| ENSSSCG00000030775 | -3,54 | 0,045 |  |  |
| ENSSSCG00000039569 | -3,51 | 0,044 |  |  |
| ENSSSCG00000031106 | -3,47 | 0,002 | *PLA2G2D* | Phospholipase A2 Group IID |
| ENSSSCG00000029239 | -3,43 | 0,012 | *MZB1* | Marginal Zone B And B1 Cell Specific Protein |
| ENSSSCG00000025523 | -3,41 | 0,002 | *COL2A1* | Collagen Type II Alpha 1 Chain |
| ENSSSCG00000035443 | -3,38 | 0,044 |  |  |
| ENSSSCG00000014977 | -3,33 | 0,015 | *PGR* | Progesterone Receptor |
| ENSSSCG00000040885 | -3,31 | 0,029 | *LY6G6E* | Lymphocyte Antigen 6 Family Member G6E |
| ENSSSCG00000007007 | -3,26 | 0,002 | *IDO1* | Indoleamine 2,3-Dioxygenase 1 |
| ENSSSCG00000014834 | -3,25 | 0,017 | *UCP3* | Uncoupling Protein 3 |
| ENSSSCG00000036983 | -3,04 | 0,045 |  |  |
| ENSSSCG00000023746 | -2,96 | 0,021 |  |  |
| ENSSSCG00000004270 | -2,93 | 0,006 | *ADGRB3* | Adhesion G Protein-Coupled Receptor B3 |
| ENSSSCG00000025042 | -2,80 | 0,015 | *ICOS* | Inducible T Cell Costimulator |
| ENSSSCG00000015332 | -2,76 | 0,010 | *PON1* | Paraoxonase 1 |
| ENSSSCG00000009239 | -2,75 | 0,022 |  | Placenta-Specific Gene 8 Protein |
| ENSSSCG00000036438 | -2,75 | 0,006 | *GPX3* | Glutathione Peroxidase 3 |
| ENSSSCG00000000419 | -2,73 | 0,003 | *RDH16* | Retinol Dehydrogenase 16 (All-Trans) |
| ENSSSCG00000006328 | -2,71 | 0,048 | *RXRG* | Retinoid X Receptor Gamma |
| ENSSSCG00000009789 | -2,51 | 0,048 | *HCAR1* | Hydroxycarboxylic Acid Receptor 1 |
| ENSSSCG00000007718 | -2,48 | 0,039 | *CLDN4* | Claudin 4 |
| ENSSSCG00000012167 | -2,48 | 0,032 | *PHEX* | Phosphate Regulating Endopeptidase Homolog X-Linked |
| ENSSSCG00000013111 | -2,42 | 0,007 | *CD6* | CD6 Molecule |
| ENSSSCG00000023522 | -2,41 | 0,015 | *TGM2* | Transglutaminase 2 |
| ENSSSCG00000027568 | -2,34 | 0,044 | *BLK* | BLK Proto-Oncogene, Src Family Tyrosine Kinase |
| ENSSSCG00000039627 | -2,33 | 0,043 | *TMEM200C* | Transmembrane Protein 200C |
| ENSSSCG00000003882 | -2,33 | 0,026 | *SLC5A9* | Solute Carrier Family 5 Member 9 |
| ENSSSCG00000034213 | -2,32 | 0,006 | *ACER2* | Alkaline Ceramidase 2 |
| ENSSSCG00000016832 | -2,28 | 0,020 | *IL7R* | Interleukin 7 Receptor |
| ENSSSCG00000028567 | -2,25 | 0,019 | *BTNL9* | Butyrophilin Like 9 |
| ENSSSCG00000039581 | -2,20 | 0,017 |  |  |
| ENSSSCG00000024166 | -2,14 | 0,005 | *SLC2A6* | Solute Carrier Family 2 Member 6 |
| ENSSSCG00000013115 | -2,13 | 0,027 | *CD5* | CD5 Molecule |
| ENSSSCG00000036892 | -2,12 | 0,034 | *GPR132* | G Protein-Coupled Receptor 132 |
| ENSSSCG00000000773 | -2,11 | 0,024 | *TUBA8* | Tubulin Alpha 8 |
| ENSSSCG00000034335 | -2,09 | 0,031 |  |  |
| ENSSSCG00000021734 | -2,03 | 0,015 | *GPR174* | G Protein-Coupled Receptor 174 |
| ENSSSCG00000037885 | -2,02 | 0,009 |  |  |
| ENSSSCG00000006736 | -2,01 | 0,002 | *CD2* | CD2 Molecule |
| ENSSSCG00000031905 | -1,98 | 0,033 | *KCNS3* | Potassium Voltage-Gated Channel Modifier Subfamily S Member 3 |
| ENSSSCG00000034689 | -1,98 | 0,027 | *GPIHBP1* | Glycosylphosphatidylinositol Anchored High Density Lipoprotein Binding Protein 1 |
| ENSSSCG00000026217 | -1,96 | 0,044 |  | Natural Killer Cells Antigen CD94 |
| ENSSSCG00000038842 | -1,92 | 0,016 | *PCDH9* | Protocadherin 9 |
| ENSSSCG00000012643 | -1,91 | 0,036 | *SH2D1A* | SH2 Domain Containing 1A |
| ENSSSCG00000007800 | -1,90 | 0,003 | *SEPT1* | Septin 1 |
| ENSSSCG00000013788 | -1,90 | 0,002 |  | Adhesion G Protein-Coupled Receptor E2-Like |
| ENSSSCG00000007552 | -1,85 | 0,034 |  | Uncharacterized LOC102162791 |
| ENSSSCG00000038003 | -1,84 | 0,015 | *ITK* | IL2 Inducible T Cell Kinase |
| ENSSSCG00000034390 | -1,82 | 0,004 | *CARD11* | Caspase Recruitment Domain Family Member 11 |
| ENSSSCG00000003608 | -1,79 | 0,004 | *LCK* | LCK Proto-Oncogene, Src Family Tyrosine Kinase |
| ENSSSCG00000040581 | -1,78 | 0,045 | *CISH* | Cytokine Inducible SH2 Containing Protein |
| ENSSSCG00000022512 | -1,77 | 0,044 | *TRDC* | T Cell Receptor Delta Constant |
| ENSSSCG00000003590 | -1,76 | 0,042 | *PTPRU* | Protein Tyrosine Phosphatase, Receptor Type U |
| ENSSSCG00000017494 | -1,76 | 0,002 | *IKZF3* | IKAROS Family Zinc Finger 3 |
| ENSSSCG00000007956 | -1,75 | 0,023 | *NLRC3* | NLR Family CARD Domain Containing 3 |
| ENSSSCG00000015093 | -1,75 | 0,024 | *CD3D* | CD3d Molecule |
| ENSSSCG00000015766 | -1,74 | 0,025 | *WDR17* | WD Repeat Domain 17 |
| ENSSSCG00000017926 | -1,74 | 0,016 | *SLC16A13* | Solute Carrier Family 16 Member 13 |
| ENSSSCG00000040140 | -1,72 | 0,026 | *CD3E* | CD3e Molecule |
| ENSSSCG00000006309 | -1,69 | 0,017 | *CD247* | CD247 Molecule |
| ENSSSCG00000012970 | -1,69 | 0,002 | *CTSW* | Cathepsin W |
| ENSSSCG00000005533 | -1,69 | 0,037 | *PTGS1* | Prostaglandin-Endoperoxide Synthase 1 |
| ENSSSCG00000013292 | -1,68 | 0,042 | *PRR5L* | Proline Rich 5 Like |
| ENSSSCG00000023320 | -1,68 | 0,034 | *CYP3A39* | Sus Scrofa Cytochrome P450 3A39 (CYP3A39), Mrna. |
| ENSSSCG00000013839 | -1,66 | 0,005 | *RASAL3* | RAS Protein Activator Like 3 |
| ENSSSCG00000008193 | -1,64 | 0,042 | *ZAP70* | Zeta Chain Of T Cell Receptor Associated Protein Kinase 70 |
| ENSSSCG00000021206 | -1,64 | 0,005 | *IL1RAP* | Interleukin 1 Receptor Accessory Protein |
| ENSSSCG00000008217 | -1,63 | 0,015 | *CD8A* | CD8a Molecule |
| ENSSSCG00000018063 | -1,62 | 0,014 |  |  |
| ENSSSCG00000029596 | -1,62 | 0,037 |  |  |
| ENSSSCG00000034763 | -1,60 | 0,048 | *IRS2* | Insulin Receptor Substrate 2 |
| ENSSSCG00000037318 | -1,54 | 0,026 | *TRABD2B* | Trab Domain Containing 2B |
| ENSSSCG00000009293 | -1,53 | 0,030 | *SPATA13* | Spermatogenesis Associated 13 |
| ENSSSCG00000033721 | -1,51 | 0,038 |  |  |
| ENSSSCG00000035256 | -1,45 | 0,002 | *SPN* | Sialophorin |
| ENSSSCG00000000672 | -1,45 | 0,002 | *CLSTN3* | Calsyntenin 3 |
| ENSSSCG00000015290 | -1,43 | 0,038 | *CDK18* | Cyclin Dependent Kinase 18 |
| ENSSSCG00000011133 | -1,43 | 0,010 | *PFKFB3* | 6-Phosphofructo-2-Kinase/Fructose-2,6-Biphosphatase 3 |
| ENSSSCG00000001472 | -1,43 | 0,040 | *SLA-DOA* | Major Histocompatibility Complex, Class II, DO Alpha |
| ENSSSCG00000000257 | -1,42 | 0,011 | *ITGB7* | Integrin Subunit Beta 7 |
| ENSSSCG00000006452 | -1,42 | 0,041 | *CD1D* | CD1d Molecule |
| ENSSSCG00000021588 | -1,41 | 0,019 | *DAPK2* | Death Associated Protein Kinase 2 |
| ENSSSCG00000036396 | -1,40 | 0,040 | *RF01956* |  |
| ENSSSCG00000017705 | -1,39 | 0,002 | *CCL5* | C-C Motif Chemokine Ligand 5 |
| ENSSSCG00000016892 | -1,37 | 0,037 | *FST* | Follistatin |
| ENSSSCG00000007470 | -1,37 | 0,047 | *RIPOR3* | RIPOR Family Member 3 |
| ENSSSCG00000014894 | -1,35 | 0,014 | *TENM4* | Teneurin Transmembrane Protein 4 |
| ENSSSCG00000006919 | -1,33 | 0,042 |  | Guanylate-Binding Protein 7 |
| ENSSSCG00000011524 | -1,32 | 0,008 | *CHL1* | Cell Adhesion Molecule L1 Like |
| ENSSSCG00000008228 | -1,32 | 0,039 | *GNLY* | Granulysin |
| ENSSSCG00000033592 | -1,32 | 0,048 | *TMC8* | Transmembrane Channel Like 8 |
| ENSSSCG00000032841 | -1,31 | 0,042 | *RF01955* |  |
| ENSSSCG00000033020 | -1,30 | 0,042 | *SLA2* | Src Like Adaptor 2 |
| ENSSSCG00000038643 | -1,27 | 0,049 | *KLF11* | Kruppel Like Factor 11 |
| ENSSSCG00000023014 | -1,26 | 0,037 | *CD300LG* | CD300 Molecule Like Family Member G |
| ENSSSCG00000012126 | -1,25 | 0,022 | *GPM6B* | Glycoprotein M6B |
| ENSSSCG00000011297 | -1,21 | 0,040 | *ABHD5* | Abhydrolase Domain Containing 5 |
| ENSSSCG00000029304 | -1,20 | 0,025 | *STEAP3* | STEAP3 Metalloreductase |
| ENSSSCG00000011849 | -1,20 | 0,010 | *TNK2* | Tyrosine Kinase Non Receptor 2 |
| ENSSSCG00000018061 | -1,19 | 0,027 |  |  |
| ENSSSCG00000032709 | -1,18 | 0,034 | *ARL4A* | ADP Ribosylation Factor Like Gtpase 4A |
| ENSSSCG00000029668 | -1,17 | 0,031 | *IL2RB* | Interleukin 2 Receptor Subunit Beta |
| ENSSSCG00000001455 | -1,15 | 0,023 | *SLA-DRB1* | MHC Class II Histocompatibility Antigen SLA-DRB1 |
| ENSSSCG00000001725 | -1,14 | 0,041 | *ADGRF5* | Adhesion G Protein-Coupled Receptor F5 |
| ENSSSCG00000039751 | -1,08 | 0,007 | *NLRC5* | NLR Family CARD Domain Containing 5 |
| ENSSSCG00000026506 | -1,06 | 0,042 | *RALGAPA2* | Ral Gtpase Activating Protein Catalytic Alpha Subunit 2 |
| ENSSSCG00000013598 | -1,05 | 0,045 | *KANK3* | KN Motif And Ankyrin Repeat Domains 3 |
| ENSSSCG00000012399 | -1,03 | 0,042 | *FOXO4* | Forkhead Box O4 |
| ENSSSCG00000024071 | -1,03 | 0,023 | *SCARF1* | Scavenger Receptor Class F Member 1 |
| ENSSSCG00000009370 | -1,02 | 0,021 | *FOXO1* | Forkhead Box O1 |
| ENSSSCG00000000665 | -0,99 | 0,034 | *RIMKLB* | Ribosomal Modification Protein Rimk Like Family Member B |
| ENSSSCG00000036223 | -0,99 | 0,042 | *ACKR1* | Atypical Chemokine Receptor 1 (Duffy Blood Group) |
| ENSSSCG00000011295 | -0,97 | 0,040 | *SNRK* | SNF Related Kinase |
| ENSSSCG00000023569 | -0,91 | 0,041 |  |  |
| ENSSSCG00000015203 | -0,90 | 0,020 | *ROBO4* | Roundabout Guidance Receptor 4 |
| ENSSSCG00000016976 | -0,86 | 0,042 | *ZNF366* | Zinc Finger Protein 366 |
| ENSSSCG00000011330 | -0,82 | 0,043 | *NBEAL2* | Neurobeachin Like 2 |
| ENSSSCG00000006087 | 0,91 | 0,018 | *CPQ* | Carboxypeptidase Q |
| ENSSSCG00000034441 | 1,01 | 0,018 | *MRGPRF* | MAS Related GPR Family Member F |
| ENSSSCG00000004705 | 1,04 | 0,034 | *MAP1A* | Microtubule Associated Protein 1A |
| ENSSSCG00000013380 | 1,09 | 0,012 | *NUCB2* | Nucleobindin 2 |
| ENSSSCG00000010312 | 1,12 | 0,042 | *PLAU* | Plasminogen Activator, Urokinase |
| ENSSSCG00000022592 | 1,15 | 0,032 | *FIBIN* | Fin Bud Initiation Factor Homolog |
| ENSSSCG00000002245 | 1,16 | 0,002 | *KATNBL1* | Katanin Regulatory Subunit B1 Like 1 |
| ENSSSCG00000029949 | 1,17 | 0,044 | *CD248* | CD248 Molecule |
| ENSSSCG00000017306 | 1,17 | 0,044 | *ITGB3* | Integrin Subunit Beta 3 |
| ENSSSCG00000020963 | 1,18 | 0,034 | *EPDR1* | Ependymin Related 1 |
| ENSSSCG00000037142 | 1,19 | 0,030 |  | Cysteine-Rich Protein 1 |
| ENSSSCG00000028076 | 1,20 | 0,017 | *ZBTB7C* | Zinc Finger And BTB Domain Containing 7C |
| ENSSSCG00000039488 | 1,22 | 0,045 | *SPON2* | Spondin 2 |
| ENSSSCG00000006335 | 1,23 | 0,037 | *RGS4* | Regulator Of G Protein Signaling 4 |
| ENSSSCG00000003876 | 1,24 | 0,002 | *CDKN2C* | Cyclin Dependent Kinase Inhibitor 2C |
| ENSSSCG00000004027 | 1,28 | 0,012 | *PDE10A* | Phosphodiesterase 10A |
| ENSSSCG00000011563 | 1,31 | 0,045 | *FANCD2* | FA Complementation Group D2 |
| ENSSSCG00000005055 | 1,33 | 0,033 | *LGALS3* | Galectin 3 |
| ENSSSCG00000009378 | 1,34 | 0,050 | *CKAP2* | Cytoskeleton Associated Protein 2 |
| ENSSSCG00000021899 | 1,35 | 0,038 |  |  |
| ENSSSCG00000037307 | 1,37 | 0,028 | *PRC1* | Protein Regulator Of Cytokinesis 1 |
| ENSSSCG00000008259 | 1,42 | 0,045 | *LRRTM4* | Leucine Rich Repeat Transmembrane Neuronal 4 |
| ENSSSCG00000000874 | 1,42 | 0,030 | *GAS2L3* | Growth Arrest Specific 2 Like 3 |
| ENSSSCG00000013517 | 1,44 | 0,040 | *UHRF1* | Ubiquitin Like With PHD And Ring Finger Domains 1 |
| ENSSSCG00000034765 | 1,44 | 0,018 |  |  |
| ENSSSCG00000014326 | 1,45 | 0,039 | *KIF20A* | Kinesin Family Member 20A |
| ENSSSCG00000037120 | 1,49 | 0,044 | *TK1* | Thymidine Kinase 1 |
| ENSSSCG00000029756 | 1,50 | 0,003 | *ADGRG2* | Adhesion G Protein-Coupled Receptor G2 |
| ENSSSCG00000040332 | 1,51 | 0,012 | *LBH* | Limb Bud And Heart Development |
| ENSSSCG00000033444 | 1,51 | 0,047 | *SPC24* | SPC24, NDC80 Kinetochore Complex Component |
| ENSSSCG00000002847 | 1,54 | 0,030 | *GPT2* | Glutamic--Pyruvic Transaminase 2 |
| ENSSSCG00000033350 | 1,57 | 0,007 | *BCHE* | Butyrylcholinesterase |
| ENSSSCG00000015091 | 1,59 | 0,022 | *MPZL2* | Myelin Protein Zero Like 2 |
| ENSSSCG00000002383 | 1,61 | 0,023 | *FOS* | Fos Proto-Oncogene, AP-1 Transcription Factor Subunit |
| ENSSSCG00000004291 | 1,61 | 0,004 | *NT5E* | 5'-Nucleotidase Ecto |
| ENSSSCG00000036801 | 1,62 | 0,030 | *C6orf132* | Chromosome 6 Open Reading Frame 132 |
| ENSSSCG00000008555 | 1,62 | 0,004 | *CGREF1* | Cell Growth Regulator With EF-Hand Domain 1 |
| ENSSSCG00000017010 | 1,63 | 0,025 | *INSYN2B* | Inhibitory Synaptic Factor Family Member 2B |
| ENSSSCG00000010325 | 1,63 | 0,001 | *KCNMA1* | Potassium Calcium-Activated Channel Subfamily M Alpha 1 |
| ENSSSCG00000016658 | 1,64 | 0,028 | *ANLN* | Anillin Actin Binding Protein |
| ENSSSCG00000004907 | 1,65 | 0,006 | *CCBE1* | Collagen And Calcium Binding EGF Domains 1 |
| ENSSSCG00000002662 | 1,67 | 0,007 | *C16orf74* | Chromosome 16 Open Reading Frame 74 |
| ENSSSCG00000002709 | 1,71 | 0,041 | *CHST6* | Carbohydrate Sulfotransferase 6 |
| ENSSSCG00000007072 | 1,73 | 0,033 | *SPTLC3* | Serine Palmitoyltransferase Long Chain Base Subunit 3 |
| ENSSSCG00000031741 | 1,73 | 0,033 | *RRM2* | Ribonucleotide Reductase Regulatory Subunit M2 |
| ENSSSCG00000025578 | 1,73 | 0,002 | *ALDH1A2* | Aldehyde Dehydrogenase 1 Family Member A2 |
| ENSSSCG00000027302 | 1,74 | 0,036 | *PKP3* | Plakophilin 3 |
| ENSSSCG00000010370 | 1,74 | 0,004 | *ANXA8* | Annexin A8 |
| ENSSSCG00000013714 | 1,81 | 0,023 |  |  |
| ENSSSCG00000022739 | 1,81 | 0,042 | *DSG2* | Desmoglein 2 |
| ENSSSCG00000009448 | 1,83 | 0,027 | *DIAPH3* | Diaphanous Related Formin 3 |
| ENSSSCG00000004886 | 1,84 | 0,036 | *CDH7* | Cadherin 7 |
| ENSSSCG00000038966 | 1,85 | 0,042 | *KRT7* | Keratin 7 |
| ENSSSCG00000008449 | 1,86 | 0,027 | *SLC3A1* | Solute Carrier Family 3 Member 1 |
| ENSSSCG00000006578 | 1,89 | 0,029 | *S100A4* | S100 Calcium Binding Protein A4 |
| ENSSSCG00000009138 | 1,89 | 0,030 | *CFI* | Complement Factor I |
| ENSSSCG00000007528 | 2,01 | 0,026 | *PHACTR3* | Phosphatase And Actin Regulator 3 |
| ENSSSCG00000017448 | 2,07 | 0,023 | *KRT14* | Keratin 14 |
| ENSSSCG00000000092 | 2,10 | 0,045 | *NPTXR* | Neuronal Pentraxin Receptor |
| ENSSSCG00000013909 | 2,11 | 0,037 | *CRLF1* | Cytokine Receptor Like Factor 1 |
| ENSSSCG00000035392 | 2,12 | 0,000 | *IGFBP2* | Insulin Like Growth Factor Binding Protein 2 |
| ENSSSCG00000033941 | 2,22 | 0,005 | *C2orf88* | Chromosome 2 Open Reading Frame 88 |
| ENSSSCG00000003508 | 2,23 | 0,017 | *KIF17* | Kinesin Family Member 17 |
| ENSSSCG00000035124 | 2,25 | 0,014 | *ILDR2* | Immunoglobulin Like Domain Containing Receptor 2 |
| ENSSSCG00000003160 | 2,25 | 0,028 | *NTF4* | Neurotrophin 4 |
| ENSSSCG00000008813 | 2,26 | 0,025 | *CORIN* | Corin, Serine Peptidase |
| ENSSSCG00000013911 | 2,30 | 0,005 | *TMEM59L* | Transmembrane Protein 59 Like |
| ENSSSCG00000021126 | 2,44 | 0,014 | *SOSTDC1* | Sclerostin Domain Containing 1 |
| ENSSSCG00000033958 | 2,46 | 0,002 | *RYR3* | Ryanodine Receptor 3 |
| ENSSSCG00000026868 | 2,54 | 0,031 | *LRRC15* | Leucine Rich Repeat Containing 15 |
| ENSSSCG00000004759 | 2,55 | 0,019 | *SPINT1* | Serine Peptidase Inhibitor, Kunitz Type 1 |
| ENSSSCG00000004191 | 2,56 | 0,038 | *MOXD1* | Monooxygenase DBH Like 1 |
| ENSSSCG00000000918 | 2,67 | 0,040 | *EPYC* | Epiphycan |
| ENSSSCG00000002883 | 2,69 | 0,020 |  |  |
| ENSSSCG00000006580 | 2,76 | 0,000 | *S100A2* | S100 Calcium Binding Protein A2 |
| ENSSSCG00000003509 | 2,77 | 0,012 | *SH2D5* | SH2 Domain Containing 5 |
| ENSSSCG00000016883 | 2,78 | 0,037 | *ISL1* | ISL LIM Homeobox 1 |
| ENSSSCG00000001832 | 2,81 | 0,038 | *ACAN* | Aggrecan |
| ENSSSCG00000006021 | 3,00 | 0,045 | *KCNV1* | Potassium Voltage-Gated Channel Modifier Subfamily V Member 1 [Source:HGNC Symbol;Acc:HGNC:18861 |
| ENSSSCG00000026780 | 3,01 | 0,005 | *EDIL3* | EGF Like Repeats And Discoidin Domains 3 |
| ENSSSCG00000003431 | 3,06 | 0,005 | *NPPB* | Natriuretic Peptide B |
| ENSSSCG00000036566 | 3,39 | 0,002 | *LY6G6C* | Lymphocyte Antigen 6 Family Member G6C |
| ENSSSCG00000038121 | 3,76 | 0,003 | *TCHH* | Trichohyalin |
| ENSSSCG00000034838 | 3,95 | 0,002 | *MAP1LC3C* | Microtubule Associated Protein 1 Light Chain 3 Gamma |
| ENSSSCG00000033927 | 4,26 | 0,001 |  |  |
