# Supplementary material for: Transcriptome analysis identifies genes involved with the development of umbilical hernias in pigs
Source: PLoS One. 2020 May 7;15(5):e0232542. doi: 10.1371/journal.pone.0232542 (PMC7205231; doi:10.1371/journal.pone.0232542)
Supplement: S3 Table — Genes in bold are upregulated in the affected pigs. (DOCX) [file pone.0232542.s003.docx]

**S3 Table:** Main biological processes of genes differentially expressed between normal and umbilical hernia-affected piglets. Genes in bold are upregulated in the affected pigs.

| **Term_ID** | **David Bioprocesses** | **Enriched genes** |
| --- | --- | --- |
| GO:0046649 | Lymphocyte activation | *NLRC3, IDO1, CD6, CD3D, IKZF3, LCK, IL7R, CD5, CD8A, MZB1,* ***LGALS3*** |
| GO:0030217 | T cell differentiation | *CD3D, LCK, IL7R, CD8A* |
| GO:0042110 | T cell activation | *NLRC3, IDO1, CD6, CD3D, LCK, IL7R, CD5, CD8A,* ***LGALS3*** |
| GO:0050670 | Regulation of lymphocyte proliferation | *IDO1, CD6, IKZF3, MZB1,* ***LGALS3*** |
| GO:0050776 | Regulation of immune response | *IDO1, LCK, BLK, IL7R, CD247,* ***SH2D1A*** |
| GO:0070489 | T cell aggregation | *NLRC3, IDO1, CD6, CD3D, LCK, IL7R, CD5, CD8A,* ***LGALS3*** |
| GO:0071593 | Lymphocyte aggregation | *NLRC3, IDO1, CD6, CD3D, LCK, IL7R, CD5, CD8A,* ***LGALS3*** |
| GO:0030098 | Lymphocyte differentiation | *CD3D, IKZF3, LCK, IL7R, CD8A* |
| GO:0070486 | Leukocyte aggregation | *NLRC3, IDO1, CD6, CD3D, LCK, IL7R, CD5, CD8A,* ***LGALS3*** |
| GO:0002684 | Positive regulation of immune system process | *IDO1, CD6, LCK, BLK, IL7R, CD5, CD247, MZB1, SH2D1A,* ***LGALS3*** |
| GO:0022409 | Positive regulation of cell-cell adhesion | *CD6, LCK, IL7R, CD5* |
| GO:0002682 | Regulation of immune system process | *IDO1, CD6, IKZF3, LCK, BLK, HCAR1, IL7R, CD5, CD247, MZB1, SH2D1A,* ***LGALS3*** |
| GO:0032943 | Mononuclear cell proliferation | *IDO1, CD6, IKZF3, IL7R, MZB1,* ***LGALS3*** |
| GO:0032944 | Regulation of mononuclear cell proliferation | *IDO1, CD6, IKZF3, MZB1,* ***LGALS3*** |
| GO:0051249 | Regulation of lymphocyte activation | *IDO1, CD6, IKZF3, LCK, IL7R, CD5, MZB1,* ***LGALS3*** |
| GO:0051251 | Positive regulation of lymphocyte activation | *CD6, LCK, IL7R, CD5* |
| GO:1903039 | Positive regulation of leukocyte cell-cell adhesion | *CD6, LCK, IL7R, CD5* |
| GO:1903037 | Regulation of leukocyte cell-cell adhesion | *IDO1, CD6, LCK, IL7R, CD5,* ***LGALS3*** |
| GO:0050870 | Positive regulation of t cell activation | *CD6, LCK, IL7R, CD5* |
| GO:0002694 | Regulation of leukocyte activation | *IDO1, CD6, IKZF3, LCK, IL7R, CD5, MZB1,* ***LGALS3*** |
| GO:0050863 | Regulation of t cell activation | *IDO1, CD6, LCK, IL7R, CD5,* ***LGALS3*** |
| GO:0050865 | Regulation of cell activation | *IDO1, CD6, IKZF3, LCK, IL7R, CD5, MZB1,* ***LGALS3*** |
| GO:0006955 | Immune response | *IDO1, CD6, LCK, BLK, IL7R, CTSW, CD247, CD8A, SH2D1A* |
| GO:0050851 | Antigen receptor-mediated signaling pathway | *LCK, BLK, CD247* |
| GO:0046651 | Lymphocyte proliferation | *IDO1, CD6, IKZF3, IL7R, MZB1,* ***LGALS3*** |
| GO:0045321 | Leukocyte activation | *NLRC3, IDO1, CD6, CD3D, IKZF3, LCK, IL7R, CD5, CD8A, MZB1,* ***LGALS3*** |
| GO:0070663 | Regulation of leukocyte proliferation | *IDO1, CD6, IKZF3, MZB1,* ***LGALS3*** |
| GO:0030198 | Extracellular matrix organization | *VIT, MMP13****, SPINT1, ACAN*** |
| GO:0001775 | Cell activation | *NLRC3, IDO1, CD6, CD3D, IKZF3, LCK, IL7R, CD5, CD8A, MZB1,* ***LGALS3*** |
| GO:0043062 | Extracellular structure organization | *VIT, MMP13****, SPINT1, ACAN*** |
| GO:0048583 | Regulation of response to stimulus | *NLRC3, IDO1, CD6, BLK, IL7R, CD247, CD8A, MZB1, SH2D1A, LCK, HCAR1, PRR5L, SPATA13****, CCBE1, NT5E, ISL1, CRLF1, LGALS3, S100A4, LRRC15*** |
| GO:0048518 | Positive regulation of biological process | *CD2, IDO1, CD3D, CD6, IKZF3, BLK, IL7R, CD5, CD247, IL1RAP, RXRG, CD8A, MZB1, SH2D1A, VIT, LCK, TGM2, PRR5L****, LGALS3, CCBE1, ISL1, CRLF1, S100A4, LRRC15*** |
| GO:0048584 | Positive regulation of response to stimulus | *IDO1, CD6, LCK, BLK, PRR5L, CD247, CD8A, SH2D1A****, CRLF1, CCBE1, ISL1, S100A4*** |
| GO:0031347 | Regulation of defense response | *IDO1, CD6, HCAR1, SH2D1A,* ***ISL1, NT5E*** |
| GO:0006952 | Defense response | *IDO1, CD6, PTGS1, LCK, BLK, HCAR1, CD8A, SH2D1A****, ISL1, NT5E*** |
| GO:0007166 | Cell surface receptor signaling pathway | *CD3D, CD6, BLK, CD247, CD8A, LCK, PTPRU****, LGALS3, LRRC15, KRT14, CCBE1, NTF4, ISL1, NPPB*** |
| GO:0007155 | Cell adhesion | *CD2, NLRC3, IDO1, CD3D, CD6, IL7R, CD5, CD8A, MPZL2, VIT, LCK, TGM2, PTPRU****, CDH7, LGALS3, DSG2, PKP3, NT5E, ACAN*** |
| GO:0098609 | Cell-cell adhesion | *CD2, NLRC3, IDO1, CD3D, CD6, IL7R, CD5, CD8A, MPZL2, LCK, PTPRU,* ***CDH7, LGALS3, DSG2, PKP3, NT5E*** |
| GO:0098602 | Single organism cell adhesion | *CD2, NLRC3, IDO1, CD3D, CD6, IL7R, CD5, CD8A, MPZL2, LCK, PTPRU,* ***LGALS3, DSG2, PKP3, NT5E*** |
| GO:0022407 | Regulation of cell-cell adhesion | *IDO1, CD6, LCK, IL7R, CD5,* ***LGALS3*** |
| GO:0045785 | Positive regulation of cell adhesion | *VIT, CD6, LCK, IL7R, TGM2, CD5* |
| GO:0030155 | Regulation of cell adhesion | *VIT, IDO1, CD6, LCK, IL7R, TGM2, CD5,* ***LGALS3*** |
| GO:0016337 | Single organismal cell-cell adhesion | *CD2, NLRC3, IDO1, CD3D, CD6, IL7R, CD5, CD8A, MPZL2, LCK, PTPRU,* ***DSG2, PKP3, NT5E, LGALS3*** |
| GO:0007159 | Leukocyte cell-cell adhesion | *NLRC3, IDO1, CD6, CD3D, LCK, IL7R, CD5, CD8A,* ***LGALS3, NT5E*** |
| GO:0022610 | Biological adhesion | *CD2, NLRC3, IDO1, CD3D, CD6, IL7R, CD5, CD8A, MPZL2, VIT, LCK, TGM2, PTPRU****, CDH7, LGALS3, LRRC15, DSG2, PKP3, NT5E, ACAN*** |
| GO:0032501 | Multicellular organismal process | *CD2, CD6, CD3D, PTGS1, IKZF3, IL7R, PGR, TGM2, NLRC3, IDO1, PHEX, IL1RAP, CD8A, COL11A2, MMP13, LCK, CLDN4****, ANLN, S100A2, CCBE1, NPTXR, ISL1, ACAN, KIF17, DSG2, EPYC, KRT14, SPINT1, CORIN, NTF4, NPPB, CRLF1*** |
| GO:0032502 | Developmental process | *IDO1, CD3D, IKZF3, IL7R, IL1RAP, PHEX, CD8A, COL11A2, MMP13, LCK, TGM2, CLDN4****, LGALS3, CRLF1, KIF17, ANLN, DSG2, EPYC, KRT14, SPINT1, CCBE1, NPTXR, NTF4, ISL1, ACAN*** |
| GO:0002376 | Immune system process | *NLRC3, IDO1, CD3D, CD6, IKZF3, BLK, IL7R, CD5, CTSW, CD247, CD8A, MZB1, SH2D1A, LCK, HCAR1****, LGALS3, ANLN*** |
| GO:0048856 | Anatomical structure development | *IDO1, CD3D, IKZF3, IL7R, IL1RAP, PHEX, CD8A, COL11A2, MMP13, LCK, TGM2, CLDN4****, LGALS3, CRLF1, KIF17, ANLN, DSG2, EPYC, KRT14, SPINT1, CCBE1, NPTXR, NTF4, ISL1, ACAN*** |
| GO:0018108 | Peptidyl-tyrosine phosphorylation | *LCK, BLK,* ***CRLF1, ISL1*** |
