# Supplementary figures and images for: Transcriptome analysis identifies genes involved with the development of umbilical hernias in pigs
Source: PLoS One. 2020 May 7;15(5):e0232542. doi: 10.1371/journal.pone.0232542 (PMC7205231; doi:10.1371/journal.pone.0232542)

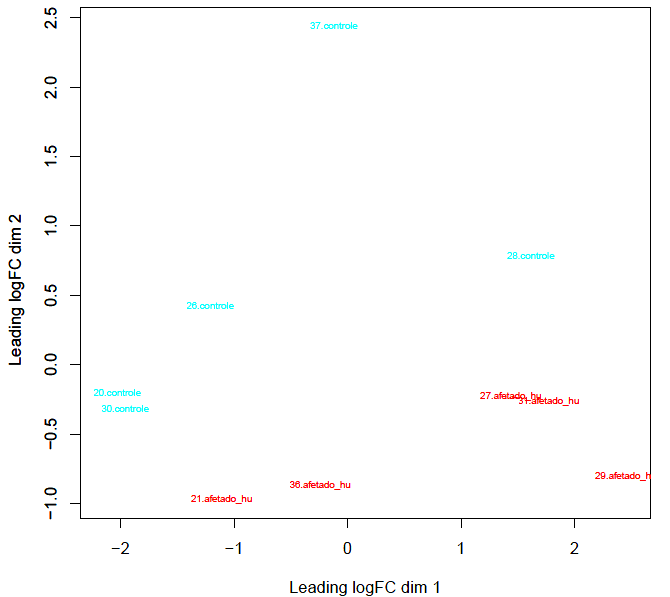

Supplement: S1 Fig — (TIF) [file pone.0232542.s004.tif]
